# *Pseudomonas aeruginosa* two-component system LadS/PA0034 regulates macrophage phagocytosis via fimbrial protein cupA1

**DOI:** 10.1128/mbio.00616-24

**Published:** 2024-05-21

**Authors:** Xiaolong Guo, Hua Yu, Junzhi Xiong, Qian Dai, Yuanyuan Li, Wei Zhang, Xiping Liao, Xiaomei He, Hongli Zhou, Kebin Zhang

**Affiliations:** 1 Clinical Medical Research Center, The Xinqiao Hospital, Army Medical University, Chongqing, China; 2 Department of orthopedics, The First Affiliated Hospital of Dalian Medical University, Dalian, China; Dartmouth College, Hanover, New Hampshire, USA

**Keywords:** two-component system, PA0034, LadS, bacterial phagocytosis by macrophages, chaperone-usher pathway pilus cupA

## Abstract

*Pseudomonas aeruginosa* is one of the most common nosocomial pathogens worldwide, known for its virulence, drug resistance, and elaborate sensor-response network. The primary challenge encountered by pathogens during the initial stages of infection is the immune clearance arising from the host. The resident macrophages of barrier organs serve as the frontline defense against these pathogens. Central to our understanding is the mechanism by which bacteria modify their behavior to circumvent macrophage-mediated clearance, ensuring their persistence and colonization. To successfully evade macrophage-mediated phagocytosis, bacteria must possess an adaptive response mechanism. Two-component systems provide bacteria the agility to navigate diverse environmental challenges, translating external stimuli into cellular adaptive responses. Here, we report that the well-documented histidine kinase, LadS, coupled to a cognate two-component response regulator, PA0034, governs the expression of a vital adhesin called chaperone-usher pathway pilus cupA. The LadS/PA0034 system is susceptible to interference from the reactive oxygen species likely to be produced by macrophages and further lead to a poor adhesive phenotype with scantily cupA pilus, impairing the phagocytosis efficiency of macrophages during acute infection. This dynamic underscores the intriguing interplay: as macrophages deploy reactive oxygen species to combat bacterial invasion, the bacteria recalibrate their exterior to elude these defenses.

**IMPORTANCE:**

The notoriety of *Pseudomonas aeruginosa* is underscored by its virulence, drug resistance, and elaborate sensor-response network. Yet, the mechanisms by which *P. aeruginosa* maneuvers to escape phagocytosis during acute infections remain elusive. This study pinpoints a two-component response regulator, PA0034, coupled with the histidine kinase LadS, and responds to macrophage-derived reactive oxygen species. The macrophage-derived reactive oxygen species can impair the LadS/PA0034 system, resulting in reduced expression of cupA pilus in the exterior of *P. aeruginosa*. Since the cupA pilus is an important adhesin of *P. aeruginosa*, its deficiency reduces bacterial adhesion and changes their behavior to adopt a planktonic lifestyle, subsequently inhibiting the phagocytosis of macrophages by interfering with bacterial adhesion. Briefly, reactive oxygen species may act as environmental cues for the LadS/PA0034 system. Upon recognition, *P. aeruginosa* may transition to a poorly adhesive state, efficiently avoiding engulfment by macrophages.

## INTRODUCTION

*Pseudomonas aeruginosa* is a ubiquitous gram-negative bacterium implicated in amounts of hospital-acquired infections, ranging from cystic fibrosis, burn/trauma-related infections, catheter-associated afflictions, diseases linked to immunodeficiency, and ventilator-associated pneumonia (1–3). Its multifaceted virulence factors and pronounced drug resistance arm *P. aeruginosa* with flexible tools to instigate a spectrum of infectious diseases (2). Another salient feature is the sensory-response regulatory matrix. The two-component systems (TCSs) provide bacteria with the ability to perceive extracellular cues, nutrient derivatives, thermal cues, pH variances, and other bactericidal signals (4–8). These intricate signal-sensing mechanisms grant *P. aeruginosa* keen insights into its external milieu, with concomitant regulatory systems directing phenotypic alterations for environmental acclimatization (9–12). With the recent identification of over 60 canonical TCS pairs within *P. aeruginosa*, many remain enigmatically characterized (13). Delving into the distinct roles of these yet-to-be-explored TCSs is paramount for a holistic comprehension of *P. aeruginosa*.

Classical TCS generally consists of two fixed components: a histidine kinase (HK) and a response regulator (RR) (11, 14). HK is equipped with a periplasmic sensing domain that specifically binds to external signals. This binding event catalyzes intracellular signal transduction, subsequently activating the kinase domain, which then auto-phosphorylates the conserved histidine residues within its dimerization and histidine phosphotransfer (DHP) domain. Notably, HK possesses phosphotransfer capabilities housed in its DHP domain, facilitating the activation of its paired RR. This is achieved by transferring phosphates from the histidine residue of HK itself onto the aspartate residues of the RR (14). Upon activation, the RR begins to exercise its regulatory functions on its downstream targets (15). Intriguingly, certain HKs may use a histidine phosphotransfer protein (Hpt) for optimal RR activation, a mechanism called intermediate phospho-relay, which culminates in nuanced gene expression regulation (16).

Infection caused by *P. aeruginosa* is generally divided into acute stage and chronic stage (17, 18). During the acute stage, the bacteria grow in a dispersed manner without biofilm formation, rendering them vulnerable to the host’s immune-mediated clearance. However, it will result in strong resistance to host clearance and antibiotic killing once the biofilm is formed in the chronic stage (19, 20). It is clear that phagocytic clearance of bacteria by macrophages (MΦs) in acute infection is critical to preventing the spread of infection (21). By the same token, it is reasonable to posit that bacterial survival and colonization during acute infections hinge significantly on mechanisms evolved by the bacteria to dodge phagocytic clearance. Therefore, it is meaningful to explore how bacteria sense the MΦs approaching and then manipulate the phenotypic transition to avoid MΦs clearance in the acute infection.

In this study, we discern that the histidine kinase, LadS, coupled to a two-component RR PA0034, positively modulates a vital adhesin named chaperone-usher pathway (CUP) pilus cupA. Notably, the LadS/PA0034 system is susceptible to interference from the reactive oxygen species (ROS), which could be originated from MΦs. This interference drives the bacteria to transition to a non-adhesive phenotype with rarely cupA pilus, thereby reducing the phagocytic efficiency of MΦs by interfering with bacterial adhesion. Briefly, the LadS/PA0034 system will be inhibited once the MΦs-derived ROS signal is sensed, and then the bacteria switch to a poorly adhesive phenotype, enhancing the ability of *P. aeruginosa* to evade phagocytic clearance during acute infection phases.

## RESULTS

### ROS inhibited the expression of putative two-component RR PA0034 in *P. aeruginosa*

**Fig 1:**
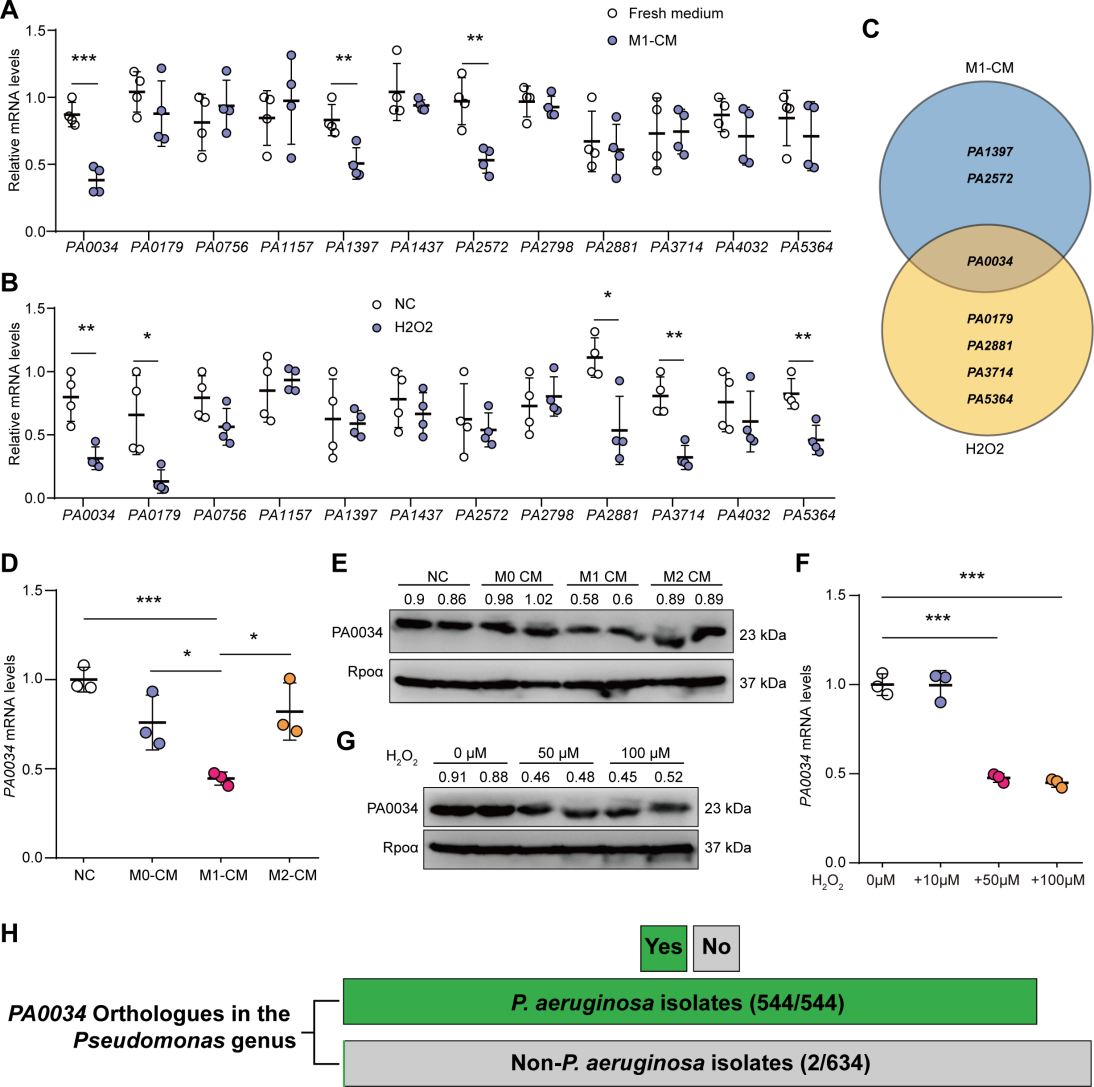
ROS inhibited the expression of two-component RR PA0034. (**A**) Reverse transcription-quantitative PCR (RT-qPCR) analysis of the RRs in PAO1 strain cultured in condition medium from pro-inflammatory MΦs (M1–CM) or fresh medium for 3 h (*n* = 4). (**B**) RT-qPCR analysis of the RRs in PAO1 strain treated with H_2_O_2_ (50 µM) for 3 h compared to normal controls (NC; *n* = 4). (**C**) Venn diagram of the changed RRs in the M1–CM-treated group or the H_2_O_2_-treated group. (**D**) RT-qPCR analysis of *PA0034* in PAO1 strain cultured in condition medium obtained from different types of MΦs (M0, M1, and M2) compared to fresh medium for 3 h (*n* = 3). (**E**) Western blotting analysis of PA0034 in PAO1 strain cultured in condition medium obtained from different types of MΦs (M0, M1, and M2) for 3 h compared to fresh medium group. RNA polymerase subunit alpha (Rpoα) as endogenous control, the intensity values relative to Rpoα were marked upon the bands. (**F**) RT-qPCR analysis of *PA0034* in PAO1 strain treated with H_2_O_2_ (10 µM, 50 µM, and 100 µM) for 3 h compared to NC group (*n* = 3). (**G**) Western blotting analysis for PA0034 in PAO1 strain treated with H_2_O_2_ (50 µM and 100 µM) for 3 h compared with NC group. Rpoα as endogenous control, the intensity values relative to Rpoα were marked upon the bands. (**H**) The distribution of *PA0034* orthologs in *P. aeruginosa* and other *Pseudomonas* genus. Green and gray bars indicate the presence and absence of *PA0034* orthologs, respectively. The data shown were mean ± SD. Statistical significance by a two-tailed unpaired *t* test is indicated: *P ≤ 0.05; **P ≤ 0.01; ***P ≤ 0.001.

For pathogen colonization, a central question is how bacteria orchestrate their lifestyle to evade host clearance in the initial stage of infection. Bacteria need to evolve proper sensory-response mechanism to avoid the phagocytosis by MΦs since the resident MΦs of barrier organs provide the first line of defense against pathogens (22). To investigate the role of unstudied TCSs in response to MΦs, the *P. aeruginosa* PAO1 strain was treated using the culture supernatant of pro-inflammatory MΦs. We identified a total of 12 undefined two-component RRs in the PAO1 genome by checking the *pseudomonas.com* and *NCBI* websites, which were listed as candidates (Fig. 1A). And the mRNA levels of *PA0034*, *PA1397*, and *PA2572* were significantly decreased under the treatment of MΦs’ supernatant compared to control group (Fig. 1A). Since the ROS was a potent bactericidal molecule unleashed by MΦs during bacterial infections (23), we further utilized the hydrogen peroxide (H_2_O_2_) to treat the PAO1 strain. The RR of PA0034, PA0179, PA2881, PA3714, and PA5364 demonstrated suppressed mRNA levels under H_2_O_2_ treatment (50 µM) relative to the control group (Fig. 1B). Notably, the expression of *PA0034* was conspicuously reduced under both treatment scenarios (Fig. 1C). The PA0034 is a putative two-component RR with unknown function before. Subsequently, the PAO1 strain was treated with culture supernatants from various MΦ subtypes (M0, untreated; M1, pro-inflammatory; M2, anti-inflammatory), indicating that only supernatants derived from M1 to MΦs could significantly downregulate the expression of *PA0034* (Fig. 1D and E). Additionally, we have treated the PAO1 strain with 10 µM, 50 µM, and 100 µM H_2_O_2_ and found that 50 µM or 100 µM, but not 10 µM group, could inhibit *PA0034* expression (Fig. 1F and G). These observations suggest that the ROS, possibly originating from MΦs, will inhibit the expression of *PA0034*.

Homologous gene analysis showed that *PA0034* orthologs were present in all of the *P. aeruginosa* isolates with complete genome currently (Fig. 1H; Fig. S1A). Furthermore, the amino acid sequences of PA0034 are consistent across all prevalent *P. aeruginosa* strains (Fig. S1B). For other *Pseudomonas* genus, only two isolates (*Pseudomonas sp. AK6U* and *Pseudomonas fluorescens NCTC10783*) were identified with *PA0034* orthologs (Fig. 1H; Fig. S1A). According to the maximum-likelihood tree analysis, these two isolates were closely related to *P. aeruginosa* (Fig. S1A). Such findings lent credence to the hypothesis that PA0034 may have a conserved functional role in *P. aeruginosa* that warrants further investigation.

### The *P. aeruginosa* PA0034 enhanced the phagocytic clearance by MΦs

**Fig 2:**
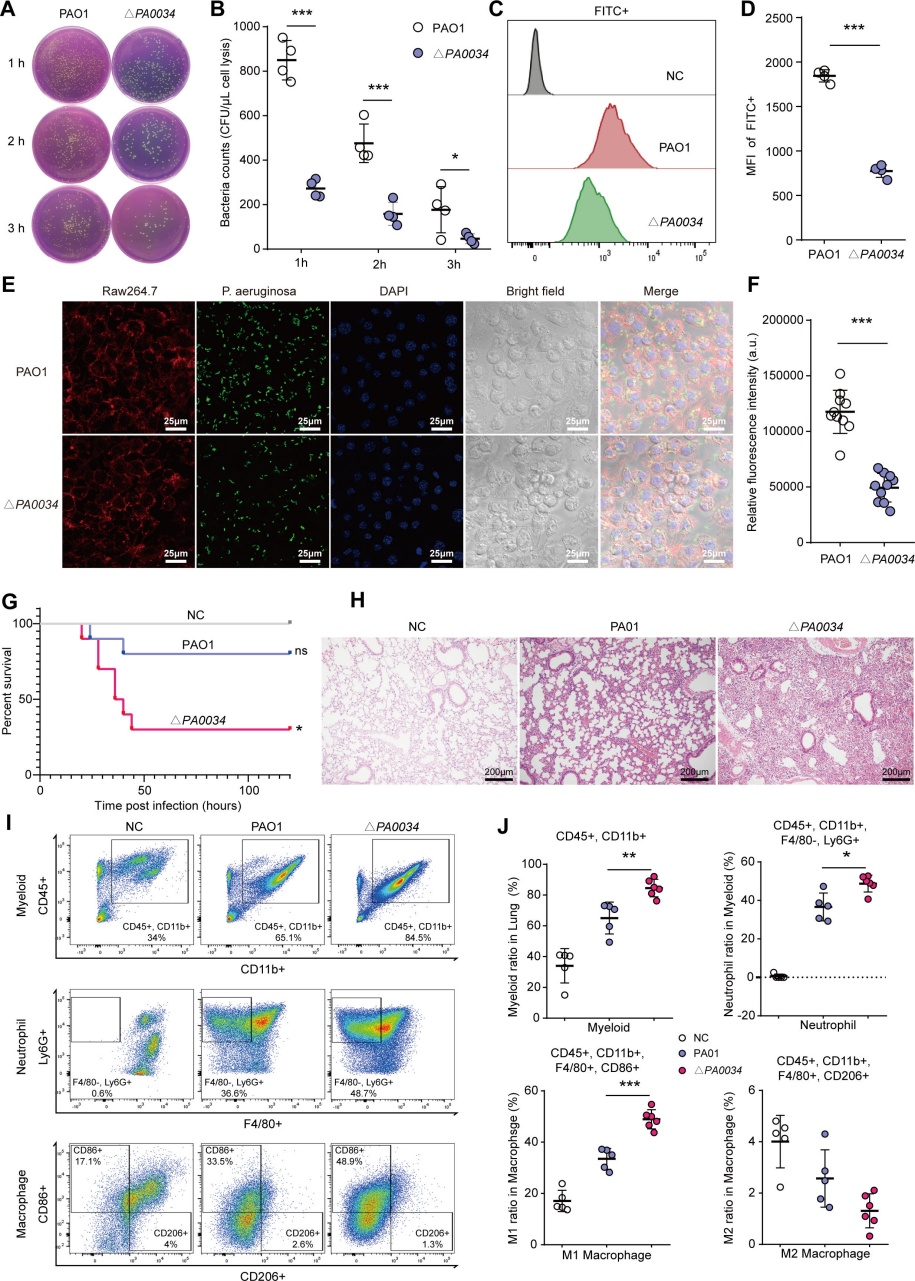
The two-component RR PA0034 increased the phagocytic clearance by MΦs. (**A and B**) The CFU counts and statistical analysis of bacteria engulfed by RAW264.7 cells between PAO1 strain and Δ*PA0034* strain (*n* = 4). Cells lysed 1 hour, 2 hours, and 3 hours post infection were plated for bacterial enumeration. (**C and D**) Fluorescence-activated cell sorting (FACS) analysis for bacterial uptake by RAW264.7 cells between PAO1 strain and Δ*PA0034* strain (*n* = 4). The histogram of FACS analysis and the mean fluorescence intensity (MFI) statistical graphs were shown. (**E**) Representative confocal images showing the bacterial uptake in PAO1 and Δ*PA0034* strain infected RAW264.7 cells. (**F**) Statistical analysis of the bacterial fluorescence intensity normalized by 4',6-diamidino-2-phenylindole (DAPI) counts. The images were acquired from triplicate experiments and analyzed by randomly selection of 10 fields of view. (**G**) The survival rate of mice was monitored during the Δ*PA0034* induced acute lung infection assay compared to the PAO1 group. Each point represents the percentage of living mice (*n* = 10). (**H**) Pathological section with hematoxylin and eosin (H&E) staining of the lung from the Δ*PA0034* induced acute lung infection mice compared to the PAO1 group (*n* = 3). (**I**) FACS analysis of the lung from the Δ*PA0034* induced acute lung infection mice compared to the PAO1 group and (**J**) statistical analysis showing the ratio of myeloid, neutrophils, M1–MΦs, and M2–MΦs in the lung tissues (NC group, *n* = 5, PAO1 group, *n* = 5, Δ*PA0034* group, *n* = 6). The data shown were mean ± SD. Statistical significance by a two-tailed unpaired *t* test is indicated: *P ≤ 0.05; **P ≤ 0.01; ***P ≤ 0.001; ns, not significant (*P* > 0.05).

Since *P. aeruginosa* senses external signals via TCS and the two-component RR PA0034 could be inhibited by ROS, we postulated that PA0034 might play a pivotal role in responding to MΦs. To ascertain whether PA0034 indeed modulated phagocytic clearance by MΦs, we constructed a *PA0034* deletion mutant based on the PAO1 strain (Δ‍*PA0034*). The colony forming units (CFU) counts obtained from MΦs uptake assay highlighted that the volume of bacteria engulfed by MΦs was considerably diminished in the Δ‍*PA0034* strain as compared to the PAO1 (Fig. 2A and B). Flow cytometric analysis further affirmed this observation that the bacterial signal (indicated by FITC+) within MΦs from the Δ*PA0034* group was markedly attenuated relative to its PAO1 counterpart (Fig. 2C and D). Moreover, fluorescence confocal imaging depicted a reduced number of internalized bacteria in the Δ*‍PA0034* treated group compared to the PAO1 group (Fig. 2E and F). Importantly, pro-inflammatory factors, such as IL1β, IL6, and TNFα, in RAW264.7 cell was significantly induced by bacterial phagocytosis in PAO1 group relative to its Δ*PA0034* counterpart (Fig. S2A). The c-Jun N-terminal kinase (JNK) and P38 kinases, not the extracellular regulated kinases (ERK), of mitogen-activated protein kinases (MAPKs) pathway, a pro-inflammatory pathway activated via bacterial phagocytosis, were markedly inhibited in RAW264.7 with Δ*PA0034* infection compared to PAO1 group (Fig. S2B). Collectively, these data underscored the essential role of two-component RR PA0034 in response to the phagocytic clearance of MΦs.

To elucidate the role of PA0034 in mediating the phagocytic clearance *in vivo*, we established an acute lung infection model of mice via tracheotomy to simulate the acute infections induced by *P. aeruginosa*. First, we found that inoculated 5 × 10^6^ CFUs with exponential PAO1 did not cause lethal pneumonia. A dose of 1.5 × 10^7^ CFUs led to sporadic fatalities (2/10), while 2.5 × 10^7^ CFUs resulted in a significant mortality rate (8/10; Fig. S2C). Striving for a balance—sufficient severity without a high fatality—we selected the 1.5 × 10^7^ CFUs dose for subsequent studies. Remarkably, the survival curves demonstrated that mice inoculated with 1.5 × 10^7^ CFUs of the Δ*PA0034* strain manifested more pronounced acute lethal pneumonia compared to the PAO1 group, which only induced modest fatalities akin to our preliminary observations (Fig. 2G). H&E staining of pathological sections of the lungs showed a more severe inflammatory infiltrate in the lungs infected with Δ*PA0034* strain relative to those exposed to PAO1 (Fig. 2H). The CFU counts further corroborated that mice infected with the Δ*PA0034* strain bore a significantly higher bacterial burden in the lung compared to the PAO1-infected counterparts (Fig. S2D and E). Furthermore, flow cytometric analysis revealed an exacerbated infiltration of neutrophils and pro-inflammatory MΦs in the lungs of mice infected with the Δ*PA0034* strain compared to the PAO1 strain (Fig. 2I and J). These data demonstrated that mice infected by Δ*‍PA0034* strain suffered from elevated bacterial loads and aggravated lung inflammation, heightening the risk of acute lethal pneumonia.

### PA0034 regulated the expression of chaperone-usher pathway pilus cupA

**Fig 3:**
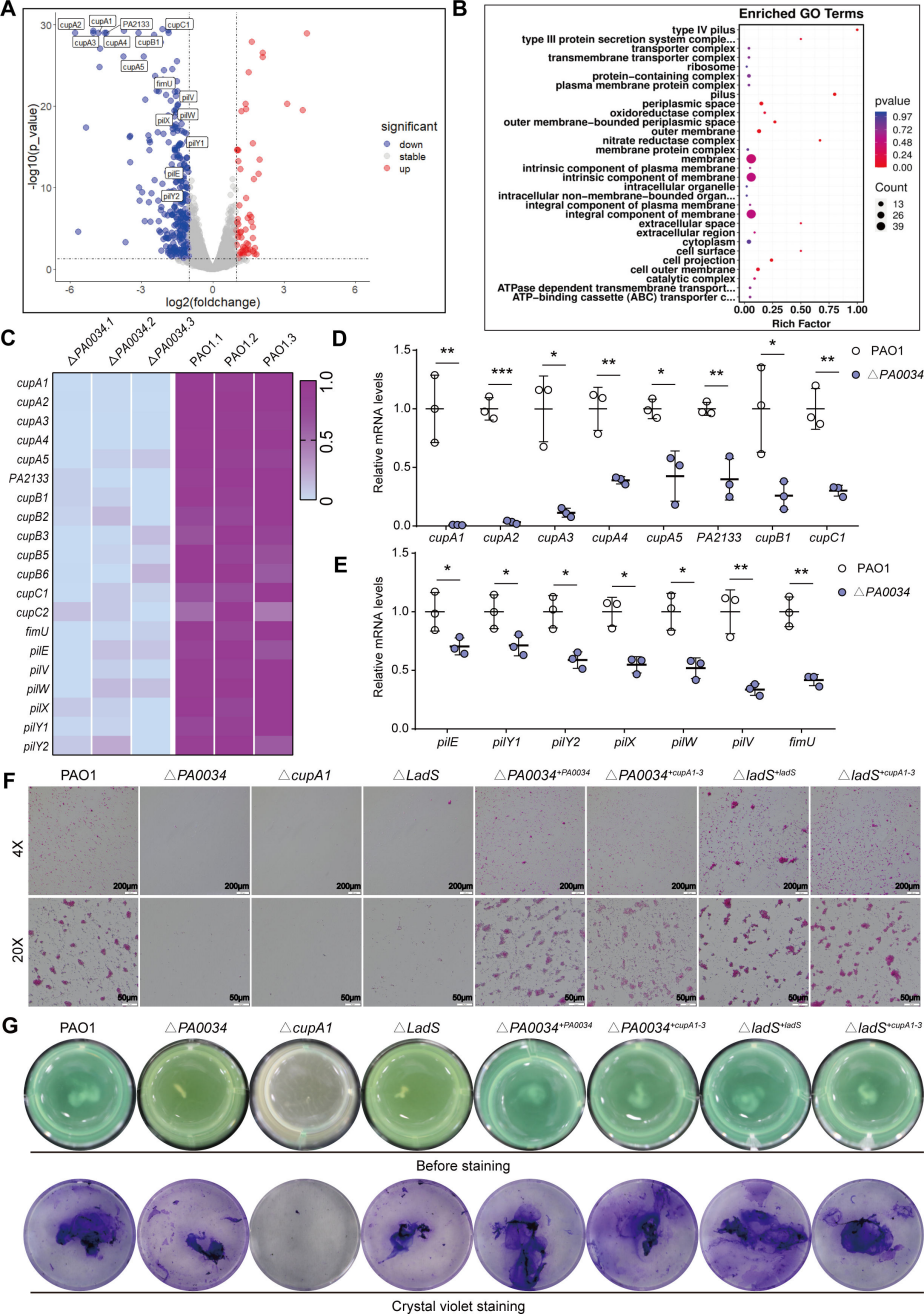
PA0034 regulates the expression of a vital adhesin called the cupA pilus. (**A**) The volcano plot of RNA-seq data showed the significantly upregulated and downregulated genes in Δ‍*PA0034* strain compared to PAO1 strain, which has been colored in red (up) and blue (down), respectively. The significantly changed pilus-related genes have been labeled on the volcano plot (*n* = 3). (**B**) The GO terms analysis with significantly changed genes of RNA-seq data in Δ‍*PA0034* strain compared to PAO1 strain (*n* = 3). (**C**) The heatmap depicted the pilus genes changed in RNA-seq data from Δ‍*PA0034* strain compared to PAO1 strain. Upregulated and downregulated genes are colored violet and gray, respectively (*n* = 3). (**D and E**) RT-qPCR analysis of pilus genes changed in RNA-seq data from Δ‍*PA0034* strain compared to PAO1 strain (*n* = 3). (**F**) The bacterial adhesive assay for Δ*PA0034*, Δ‍‍*cupA1*, Δ‍*ladS*, Δ‍*PA0034^+PA0034^*, Δ‍*PA0034^+cupA1-3^*, Δ‍*ladS^+ladS^*, and Δ‍*ladS^+cupA1-3^* strains compared to PAO1 strain was performed on 24-well polystyrene plates (*n* = 3). (**G**) The biofilm formation for Δ*PA0034*, Δ‍‍*cupA1*, Δ‍*ladS*, Δ‍*PA0034^+PA0034^*, Δ‍*PA0034^+cupA1-3^*, Δ‍*ladS^+ladS^*, and Δ‍*ladS^+cupA1-3^* strains compared to PAO1 strain was detected by crystal violet staining (*n* = 3). The data shown were mean ± SD. Statistical significance by a two-tailed unpaired *t* test is indicated: *P ≤ 0.05; **P ≤ 0.01; ***P ≤ 0.001.

Our data suggested that the Δ*‍PA0034* strain was less likely to be cleared *in vitro* and *in vivo*. Subsequent RNA-seq analysis showed that a total of 283 downregulated and 79 upregulated genes [log2(fold change) ≥ 1 and false discovery rate (FDR) ≤ 0.05] occurred in Δ‍*PA0034* compared to PAO1 strain. The gene ontology (GO) terms analysis and volcano plot shed light on the pronounced alterations in pilus components in the Δ‍*PA0034* strain (Fig. 3A and B). The downregulated pilus genes in the Δ*PA0034* strain were displayed by heat map (Fig. 3C) and further validated with RT-qPCR (Fig. 3D and E). Notably, among these pilus genes, the *cupA* cluster had the most predominant alterations in Δ*‍PA0034* strain (Fig. 3A; Table S2). Studies have shown that CUP pili are among the most widely distributed and best-characterized adhesins in gram-negative bacteria (24). Confocal imaging indicated that the captured/adherent living bacteria on MΦs were substantially reduced in the Δ*PA0034* group compared to PAO1 group (Fig. S3A). The cupA pilus is the major CUP pilus in *P. aeruginosa* (25). In order to assess the change in the adhesive capacity of bacteria which may have a positive influence on the phagocytic clearance of MΦs, the *cupA1* mutant strain (Δ*cupA1*) was constructed, and the bacterial adhesion assay was performed. A significant reduction in bacterial adhesion (Fig. 3F) and higher densities of bacteria in the supernatant medium (Fig. S3B) were found in the Δ*PA0034* and Δ*cupA1* strains compared to PAO1 strain. Importantly, bacterial adhesion was extremely low in the absence of cupA1, possibly contributing to low phagocytosis efficiency (Fig. 3F). Furthermore, adhesive weakness in the early stage leads to the inhibition of biofilm synthesis in later stages, as found in Δ*PA0034* and Δ*cupA1* strains compared with the PAO1 strain (Fig. 3G; Fig. S3C).

Given that most two-component RRs have a role in transcriptional regulation of target DNA, we postulated that PA0034 may have a role in *cupA* genes expression. The binding abundance of PA0034 on the promoter region of the *cupA* gene set was assessed via the chromatin immunoprecipitation coupled qPCR (CHIP-qPCR) assay. A schematic demarcated the primer locations on the *cupA1* promoter region (Fig. S3D). We found that two binding sites displayed pronounced PA0034 association. Of these, the target 10 site demonstrated superior enrichment compared to target 7 (Fig. S3E and F).

### Fimbrial protein cupA1 promoted bacterial phagocytosis by MΦs

**Fig 4:**
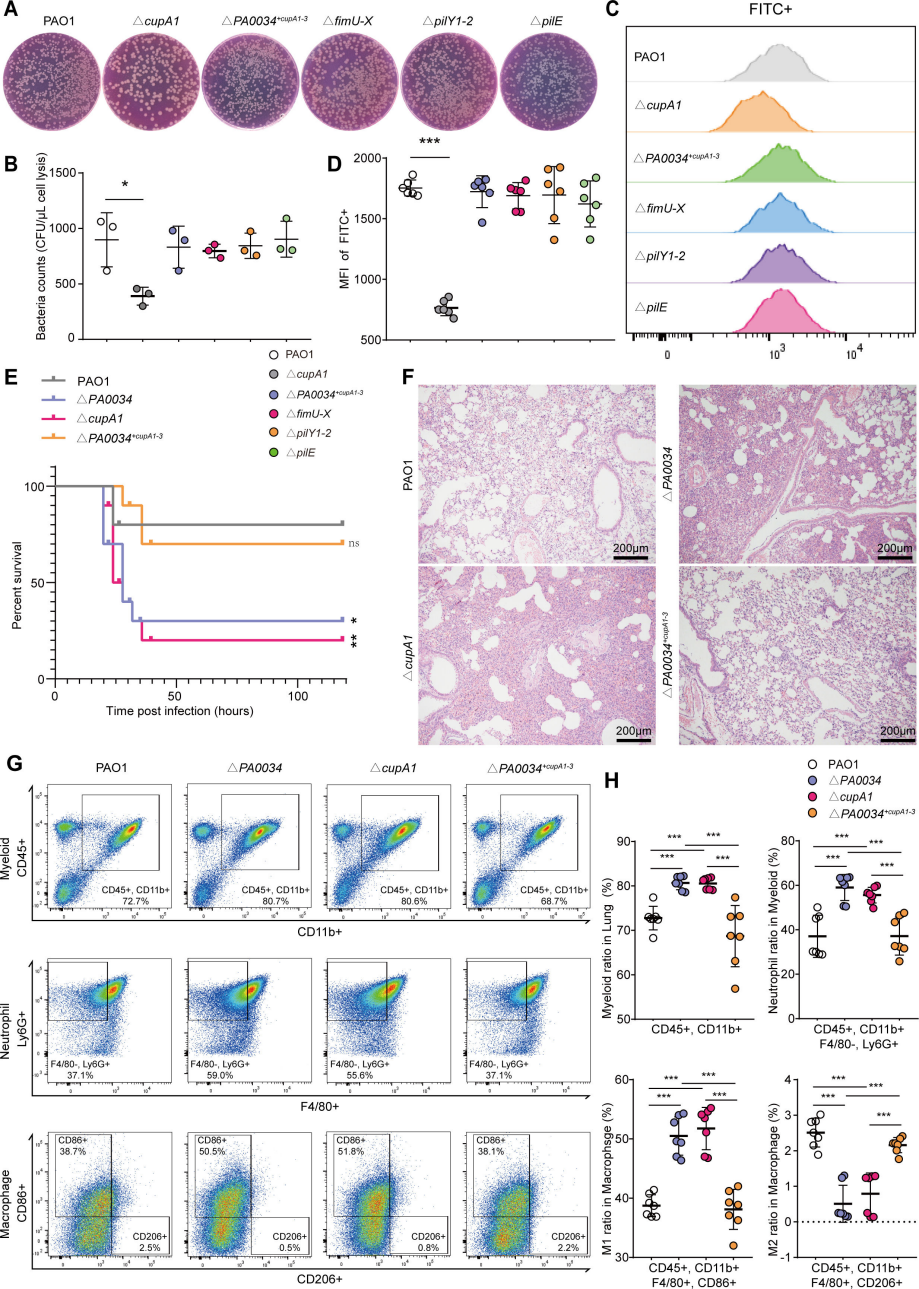
Fimbrial protein cupA1 increased bacterial phagocytosis by MΦs. (**A and B**) The CFU counts and statistical analysis of bacteria engulfed by RAW264.7 cells among the PAO1, Δ‍*cupA1*, Δ‍‍*‍PA0034^+cupA1-3^*, Δ‍*fimU-X*, Δ‍*pilY1-2*, and Δ‍*pilE* infected group (*n* = 3). (**C and D**) FACS analysis for bacterial uptake by RAW264.7 cells infected with PAO1, Δ‍*cupA1*, Δ‍‍*‍PA0034^+cupA1-3^*, Δ‍*fimU-X*, Δ‍*pilY1-2*, and Δ‍*pilE* strains (*n* = 6). The histogram of FACS analysis (**C**) and the mean fluorescence intensity (MFI) statistical graphs (**D**) were shown. (**E**) Mice infected with PAO1, Δ‍*PA0034*, Δ‍*cupA1*, and Δ‍‍*PA0034^+cupA1-3^* strains with 1.5*107 CFU via tracheotomy. The survival rates were monitored, and each point represents the percentage of living mice (*n* = 10). (**F**) Pathological section with H&E staining of the lung from the PAO1, Δ‍*PA0034*, Δ‍*cupA1*, and Δ‍‍*PA0034^+cupA1-3^* strains-induced acute lung infection mice were shown (*n* = 3). (**G and H**) FACS analysis of the lung from the PAO1, Δ‍*PA0034*, Δ‍*cupA1*, and Δ‍‍*PA0034^+cupA1-3^* strains-induced acute lung infection mice, and statistical analysis showing the ratio of myeloid, neutrophils, M1–MΦs, and M2–MΦs in the lung tissues (*n* = 7). The data shown were mean ± SD. Statistical significance by a two-tailed unpaired *t* test is indicated: *P ≤ 0.05; **P ≤ 0.01; ***P ≤ 0.001; ns, not significant (*P* > 0.05).

We speculated that bacteria, which do not adhere to settle but tend to live in a planktonic state, would reduce the efficiency of phagocytosis of bacteria by MΦs. We constructed six mutant strains based on the altered adhesion genes in Δ*PA0034* strain, including Δ*cupA1*, Δ*fimU-X*, Δ*pilY1-2*, Δ‍*pilE*, Δ‍*cupB1*, and Δ*cupC1*. CFU counts after phagocytosis assay revealed a pronounced decrease in internalized bacteria in the Δ*cupA1* group to the PAO1 group (Fig. 4A and B). However, such reductions were not present in the Δ*fimU-X*, Δ*pilY1-2*, Δ‍*pilE*, Δ*cupB1*, and Δ*cupC1* groups (Fig. 4A and B; Fig. S5A and B). Flow cytometric analysis echoed these findings, with the diminished signal of internalized bacteria in the Δ*cupA1*, no other mutants, compared to PAO1 group (Fig. 4C and D; Fig. S5C and D). The activation of JNK and *P38* kinases, but not the ERK kinases, was markedly reduced in MΦs infected with Δ*cupA1* compared to the PAO1 group (Fig. S2B). In order to ascertain whether the phagocytic inhibition was mediated by the deficiency of cupA pilus, the Δ*PA0034* strain was engineered to reacquire the cupA pilus by backfilling three core pilus genes (*cupA1-3*). Eventually, the validity of cupA pilus backfilling strain was validated by the transmission electron microscope (TEM) detection (Fig. S4A). Both CFU counts and flow cytometric analysis verified that swallowed bacteria by Raw264.7 in Δ*PA0034^+cupA1-‍3^* group were similar to PAO1 group (Fig. 4A through D). Furthermore, confocal imaging reconfirmed that internalized bacteria in Δ*cupA1* and Δ‍*PA0034* groups were decreased compared to the PAO1 group, while it was enhanced in Δ*PA0034^+cupA1-‍3^* compared to the Δ*PA0034* group (Fig. S5E and F). Cumulatively, these findings suggested that the fimbrial protein cupA1 promoted bacterial phagocytosis by MΦs.

To investigate the role of *cupA1*-mediated phagocytic clearance *in vivo*, mice were inoculated with these mutants to induce acute lung infection. Evidently, the mortality rates observed in the Δ‍‍*cupA1* and Δ‍*PA0034* cohorts mirrored each other, both surpassing the severity seen in the PAO1 group (Fig. 4E). Δ*PA0034^+cupA1-3^* strain caused a mildly lethal infection like the PAO1 strain (Fig. 4E). Pathological sections of the lungs showed aggravated inflammation in mice infected with either Δ*PA0034* or Δ*cupA1* strains, but not *PA0034^+cupA1-‍3^* strain, compared to the PAO1 strain (Fig. 4F). Flow cytometric analysis further underscored these findings, unveiling augmented neutrophil and pro-inflammatory MΦ proportions in the lungs of Δ*‍PA0034* and Δ‍*cupA1* infected mice compared to the PAO1 group (Fig. 4G and H). The neutrophil and MΦ proportions in the Δ*PA0034^+cupA1-‍3^* infected mice were dialed down, mirroring the PAO1 cohort (Fig. 4G and H). As expected, the bacterial loads of lung in the Δ*PA0034* and Δ*cupA1* group, not in the Δ‍*PA0034^+cupA1-‍3^* group, were significantly higher compared to the PAO1 group (Fig. S5G and H). These results suggested that *P. aeruginosa* lacking the fimbrial protein cupA1 was less likely to be eliminated by the host, which favored bacterial proliferation and led to more severe infections.

### Histidine kinase LadS activated PA0034 and promoted cupA pilus expression

**Fig 5:**
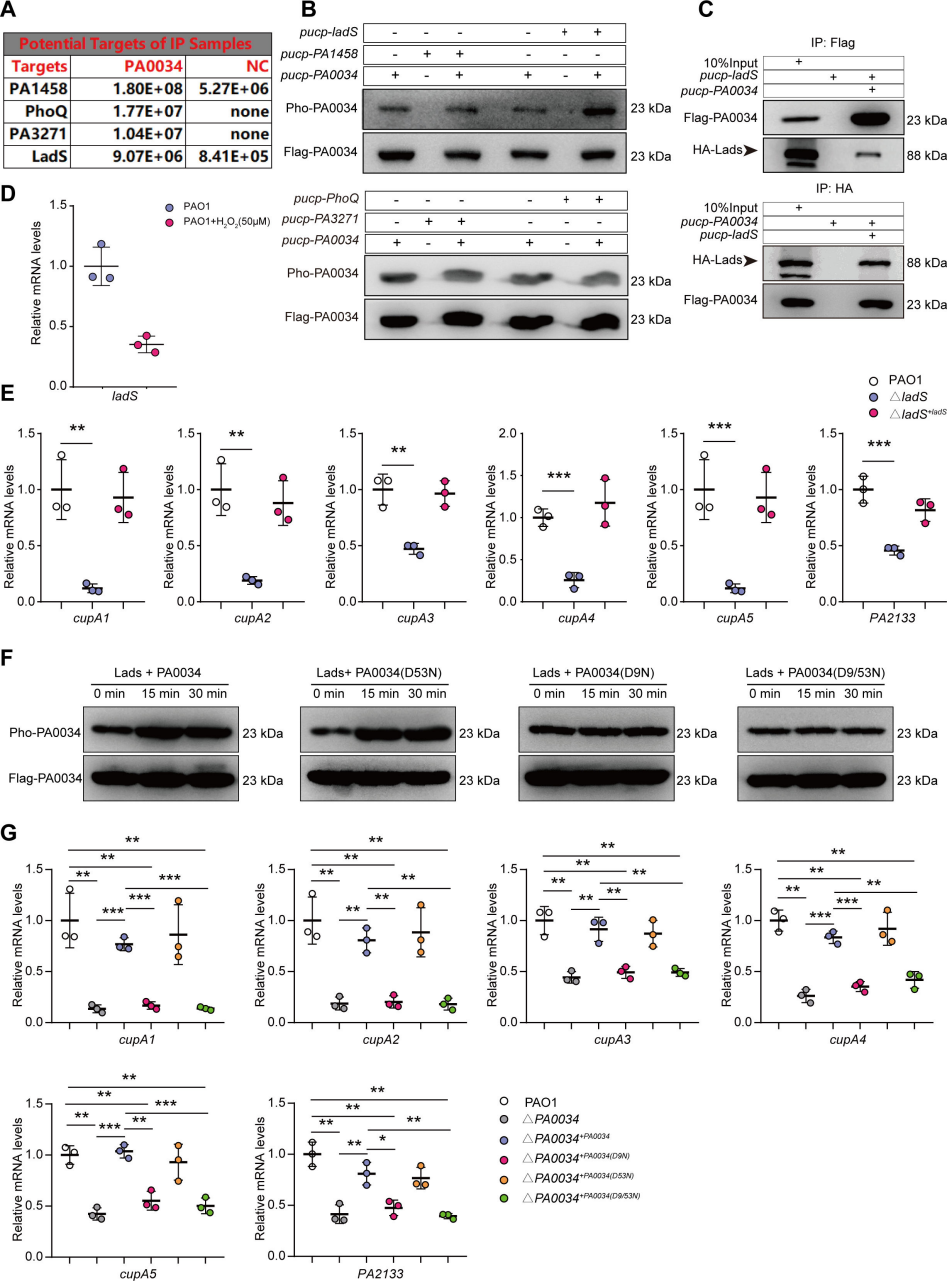
Two-component HK LadS activated PA0034 and promoted the expression of cupA pilus. (**A**) Four HKs with high abundance identified by the PA0034 co-IP coupling MS sequencing. (**B**) The phosphotransfer assay *in vitro* to evaluate the phosphorylation of PA0034 in flag-PA0034 purified sample when incubated with different HKs. The anti-Flag or anti-phosphorylation (anti-Pho) was detected by western blotting with the same membrane through stripping and re-probing process. (**C**) Co-IP experiments to evaluate the proteins interaction between flag-PA0034 and HA-LadS. (**D**) RT-qPCR analysis of *ladS* mRNA levels in PAO1 strain treated with H_2_O_2_ (50 µM) compared to a blank control (*n* = 3). (**E**) RT-qPCR analysis of the *cupA* gene set (*cupA1*, *cupA2*, *cupA3*, *cupA4*, *cupA5*, and *PA2133*) in PAO1, Δ*ladS*, and Δ*ladS^+ladS^* strains (*n* = 3). (**F**) The phosphotransfer assay *in vitro* to evaluate the phosphorylation of different PA0034 mutant in anti-flag purified samples when incubated with LadS for 15 min or 30 min. (**G**) RT-qPCR analysis of the expression of *cupA* gene set in PAO1, Δ*PA0034*, Δ*‍PA0034^+PA0034^*, Δ‍‍*PA0034^+PA0034(D9N)^*, Δ‍*PA0034^+PA0034(D53N)^*, and Δ*‍‍‍PA0034^+PA0034(D9/53N)^* strains (*n* = 3). The data shown were mean ± SD. Statistical significance by a two-tailed unpaired *t* test is indicated: *P ≤ 0.05; **P ≤ 0.01; ***P ≤ 0.001.

Given that each two-component RR had a cognate HK responsible for its activation, we exogenously expressed a Flag tagged *PA0034* allele in the Δ*PA0034* strain. The potential interacting proteins of PA0034 were evaluated by co-immunoprecipitation (co-IP) experiments coupled with mass spectrometry (MS) analysis. The four HKs (PA1458, PA3271, PhoQ, and LadS) with high abundance in MS analysis were selected for further investigation (Fig. 5A). The increased phosphorylation of flag-PA0034 was verified by the phosphotransfer assay when added the samples with the exogenously expressed LadS, but not other HKs (Fig. 5B). The protein interaction between LadS and flag-‍PA0034 was verified via western blotting (Fig. 5C). Additionally, the mRNA expression of *ladS* was inhibited by H_2_O_2_ treatment (Fig. 5D), and the phosphorylation of PA0034 was also decreased under 50 µM H_2_O_2_ treatment (Fig. S5I). To determine whether the deletion LadS affected the expression of *cupA* gene set, we deleted the histidine kinase domain of *ladS* in the PAO1 strain (Δ*‍ladS*). The Δ*‍ladS* strain displayed significantly reduced expression of *cupA* gene set compared to the PAO1 strain and the replenished *ladS* (Δ*‍LadS^+LadS^*) allele restored it (Fig. 5E). These findings indicated that LadS was responsible for the phosphorylation of PA0034 and was essential for the expression of *cupA* gene set. Furthermore, a significant reduction in bacterial adhesion and higher densities of bacteria in the supernatant medium was found in the Δ*ladS* strain compared to PAO1 strain (Fig. 3F; Fig. S3B). Adhesive weakness in the early stage leads to the reduction of biofilm synthesis in later stages, as found in Δ*ladS* strains compared with PAO1 strain (Fig. 3G; Fig. S3C).

Generally, a conserved aspartate site on RR is typically subject to phosphorylation by its paired HK (26, 27). Two aspartate residues (D9 and D53) that may activate the PA0034 protein were found through the uniprot.org website. To discern which site was responsible for the PA0034 activation, the alleles of *flag-PA0034* modified in *D9* or *D53* by substituting the aspartate residue to asparagine residues (*D9N*, *D53N*, and *D9/53N*) were exogenously expressed in the Δ*PA0034* strain, hereafter called Δ*‍PA0034^+PA0034(D9N)^*, Δ*PA0034^+PA0034(D53N)^*, and Δ*PA0034^+PA0034(D9/53N)^* strain. We found that the phosphorylation of PA0034 was inhibited by *D9N* or *D9/53N* mutants, but not in *D53N* mutant (Fig. 5F). This finding indicated that LadS specifically recognized the D9 residue for phosphorylation. Additionally, the expression of *cupA* gene set was significantly diminished in the Δ*PA0034^+PA0034(D9N)^* and Δ*PA0034^+PA0034(D9/53N)^* strains compared to the PAO1 strain, but not in theΔ*PA0034^+PA0034^* and Δ*PA0034^+PA0034(D53N)^* strains (Fig. 5G). This highlights the role of the D9 residue in PA0034 activation, which was crucial for the expression of the *cupA* gene set.

Furthermore, the LadS is a hybrid histidine kinase that could use an intermediate phospho-relay to activate the paired RR. By consulting the PAO1 genome, the gene encoded next to *PA0034* is a phospho-relay, called *hptC*. Therefore, the *hptC* gene may play an essential role in the LadS/PA0034 network, so the *hptC* mutant was constructed to verify the effect of HptC on the LadS/PA0034 network. The CFU counts and flow cytometric analysis from the MΦ uptake assays revealed that fewer bacteria engulfed in the Δ*hptC* infected group than the PAO1 group (Fig. S5A through D). These findings indicated that hptC may be a paired Hpt for PA0034 and responsible for its activation.

### *ladS* mutation and PA0034 inactivation inhibited the bacterial phagocytosis of MΦs

**Fig 6:**
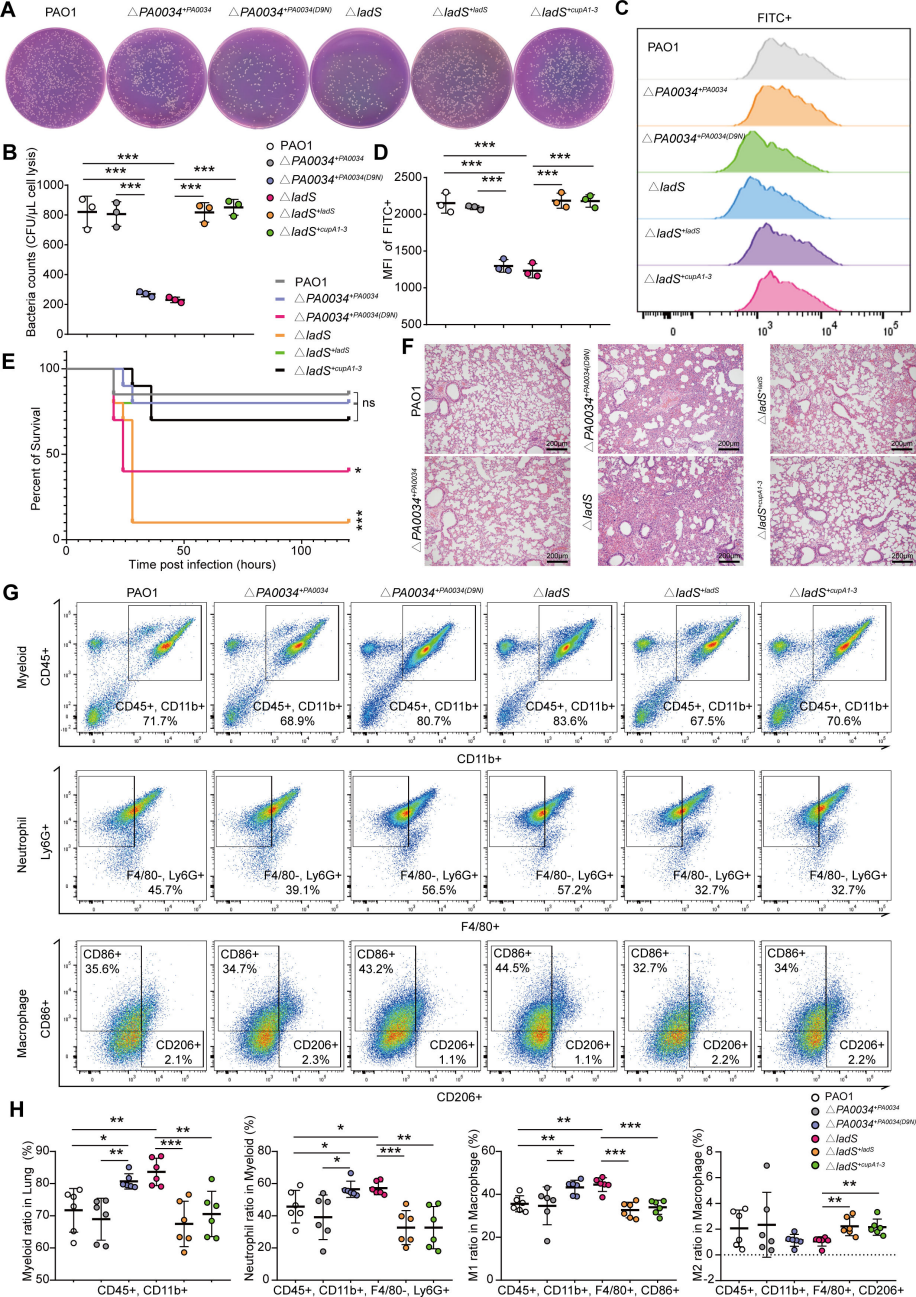
LadS and PA0034 activation promoted bacterial phagocytosis by MΦs. (**A and B**) The CFU counts and statistical analysis of bacteria engulfed by RAW264.7 cells in PAO1, Δ*‍‍PA0034^+PA0034^*, Δ‍‍*PA0034^+PA0034(D9N)^*, Δ*‍ladS*, Δ‍*ladS^+ladS^*, and Δ*ladS^+cupA1-3^* infected groups (*n* = 3). Cell lysates were plated for bacterial enumeration following serial dilution. (**C and D**) FACS analysis for bacterial uptake by RAW264.7 cells in PAO1, Δ*‍‍PA0034^+PA0034^*, Δ‍‍*PA0034^+PA0034(D9N)^*, Δ*‍ladS*, Δ‍‍*ladS^+ladS^*, and Δ‍*ladS^+cupA1-3^* infected groups. The histogram of FACS analysis and the mean fluorescence intensity (MFI) statistical graphs were shown. (**E**) The survival rate of mice was monitored during the PAO1, Δ*‍‍PA0034^+PA0034^*, Δ‍‍*PA0034^+PA0034(D9N)^*, Δ*‍ladS*, Δ‍‍*ladS^+ladS^*, and Δ‍*ladS^+cupA1-3^* strains-induced acute lung infection assay. Each point represents the percentage of living mice (*n* = 10). (**F**) Pathological section with H&E staining of the lung from the PAO1, Δ*‍‍PA0034^+PA0034^*, Δ‍‍*PA0034^+PA0034(D9N)^*, Δ*‍ladS*, Δ‍‍*ladS^+ladS^*, and Δ‍*ladS^+cupA1-3^* strains-induced acute lung infection mice (*n* = 3). (**G and H**) FACS analysis of the lung from PAO1, Δ*‍‍PA0034^+PA0034^*, Δ‍‍*PA0034^+PA0034(D9N)^*, Δ*‍ladS*, Δ‍‍*ladS^+ladS^*, and Δ‍*ladS^+cupA1-3^* strains-induced acute lung infection mice, and statistical analysis showing the ratio of myeloid, neutrophils, M1–MΦs, and M2–MΦs in the lung tissues (*n* = 6). The data shown were mean ± SD. Statistical significance by a two-tailed unpaired *t* test is indicated: *P ≤ 0.05; **P ≤ 0.01; ***P ≤ 0.001; ns, not significant (*P* > 0.05).

Both LadS and the PA0034 activation were responsible for the expression of the *cupA* gene set. However, whether the phagocytic efficiency of MΦs was compromised by either the *ladS* mutation or PA0034 inactivation remained ambiguous. The CFU counts from MΦ uptake assays revealed that the phagocytic efficiency with Δ*‍ladS* strain and Δ*PA0034^+PA0034 (D9N)^* strain was decreased markedly compared to PAO1 group, yet this effect was reversed when the Δ*‍ladS* strain compensated with *ladS* allele (Δ*‍ladS^+ladS^*) or *cupA1-3* allele (Δ*‍ladS^+cupA1-3^*) and the Δ‍*PA0034* strain compensated with *PA0034* allele (Δ*PA0034^+PA0034^*; Fig. 6A and B). As expected, flow cytometric analysis showed that the bacterial signal engulfed by MΦs was significantly decreased in Δ*‍PA0034^+PA0034‍(D9N)^* and Δ*‍ladS* groups compared to the PAO1 group, and the Δ*‍PA0034^+PA0034^*, Δ*‍ladS^+ladS^*, and Δ*ladS^+cupA1-3^* groups remained akin to the PAO1 group (Fig. 6C and D). Fluorescence confocal imaging also showed consistent conclusions that the mutation of *ladS* and inactivation of PA0034 impaired bacterial phagocytosis by MΦs (Fig. S6A and B). Additionally, to assess the influence of bacterial adhesion on the efficiency of bacteria capture by macrophages while excluding interference with the endocytosis effect of the macrophage, RAW264.7 cells were pre-incubated with the actin cytoskeletal depolymerizing agent cytochalasin D (cytD) to inhibit endocytosis, and bacterial phagocytosis assay indicated that the ability of macrophages to catch bacteria in Δ*PA0034*, Δ*ladS*, or Δ*cupA1* strains was significantly inhibited compared to PAO1 strain (Fig. S6C and D). These results suggested that PA0034, ladS, or cupA1 knockout led to the bacteria not adhering to settle but tend to live in a planktonic state, which reduces the efficiency of bacteria capture by macrophages.

Next, using the acute lung infection model of mice, we observed that the survival rate in Δ*‍PA0034^+PA0034‍ (D9N)^* and Δ*ladS* infected groups was significantly lower than PAO1 group, while mice infected by Δ*‍PA0034^+PA0034^*, Δ*ladS^+ladS^*, and Δ*ladS^+cupA1-3^* strain resulted in mild lethality akin to the PAO1 strain (Fig. 6E). Pathological sections of the lung showed more severe acute infection in Δ*‍PA0034^+PA0034‍ (D9N)^* and Δ*ladS* infected mice than PAO1 infected mice, while the Δ*‍PA0034^+PA0034^*, Δ*‍ladS^+ladS^*, and Δ*‍ladS^+cupA1-3^* strains caused a mild inflammation like the PAO1 strain (Fig. 6F). Flow cytometric analysis displayed higher proportions of neutrophils and pro-inflammatory MΦs in Δ*‍PA0034^+PA0034‍ (D9N)^* and Δ*ladS* infected mice than the PAO1 group, while the inflammation has been alleviated in Δ*‍PA0034^+PA0034^*, Δ*‍ladS^+ladS^*, and Δ*‍ladS^+cupA1-3^* infected group to an extent comparable to PAO1 group (Fig. 6G and H). Furthermore, CFU counts from the lung tissues illustrated that the bacterial loads in Δ*‍PA0034^+PA0034‍ (D9N)^* and Δ*‍ladS* infected group were higher than PAO1 group, and the bacterial loads in Δ*‍PA0034^+PA0034^*, Δ*ladS^+ladS^*, and Δ*‍ladS^+cupA1-3^* infected groups were equivalent to the PAO1 group (Fig. S6E and F). These results implied that mutation of *ladS* or inactivation of PA0034 both impaired the bacterial clearance of host in the acute lung infection of mice.

## DISCUSSION

It is well-established that phagocytic clearance by MΦs represents a crucial mechanism through which hosts eliminate bacteria, preventing further exacerbation of infections in the early stages (21, 28). Studies for understanding how bacteria evade host immune system clearance during acute infections remain a priority. In this study, our findings indicated that the culture supernatant of pro-inflammatory MΦs can significantly inhibit the expression of PA0034, a putative two-component RR in PAO1 strain. Further study showed that the PA0034 was a cognate RR of LadS that involved in the regulation of cupA pilus. The signal molecule in the culture supernatant of MΦs recognized by *P. aeruginosa* was ROS, potent bactericidal agents produced by MΦs upon bacterial infection. However, the specific mechanism by which ROS regulates LadS/PA0034 system expression remains poorly understood. According to our data, ROS inhibited the expression of LadS, and LadS can activate PA0034. Two-component RR may display intrinsic feedback that regulates the expression of genes themselves (15, 29). Therefore, the decreased expression of LadS and impaired activation of PA0034 may lead to downregulation of PA0034. However, ROS also has a widespread impact on bacterial physiology, and a published RNA-seq data suggested that ROS signaling disrupts the expression of a wide range of genes in *P. aeruginosa*, including a number of regulatory factors (30). There may be a potential regulator or system that responds to ROS signaling directly and is specifically responsible for the regulation of LadS/PA0034. Further research is still needed to investigate the mechanisms of how ROS signaling regulates the expression of the LadS/PA0034 system.

Studies show that CUP pilus is among the most widely distributed and best-characterized adhesins in gram-negative bacteria (24). The cupA pilus is the major CUP pilus in *P. aeruginosa* (25). Additional chaperone-usher pathway pilus (cupB and cupC) also has been identified in PAO1 genome with a lower expression that does not appear to play a role in adhesion under the conditions where the cupA is present (25). We also found that cupB and cupC pili were dispensable for bacterial phagocytosis by MΦs (Fig. S5A through D).

The cupA pilus was essential for bacterial adhesion and mediated the phagocytosis of MΦs. It is currently considered that CUP systems typically consist of at least three components: a major pilin subunit, a periplasmic chaperone that stabilizes the pilin prior to assembly, and an outer membrane usher pore protein responsible for translocation and assembly of the pilin (31, 32). The chaperone facilitates the folding of pilus subunits, prevents them from polymerizing in the periplasm, and targets them to the usher (33). The usher acts as a pilus assembly platform, recruiting chaperone–subunit complexes from the periplasm, coordinating their assembly into a pilus, and secreting that pilus through the usher pore (34, 35). We have successfully backfilled the cupA pilus with an alternative sequence containing the core components (cupA1-3) of cupA pilus. Although previous studies have shown this approach to be theoretically feasible, it is worth considering that several components remain unexpressed in this approach. One possible explanation is that the expression and supply of cupA4, cupA5, and PA2133 in PAO1 were naturally low. As the expression of *cupA* cluster in ΔPA0034 strain, the most reduced gene in *cupA* cluster was seen in the *cupA1-3* genes, whereas the *cupA4*, *cupA5*, and *PA2133* were still expressed at a readily detectable level (Fig. 4D), which offers the possibility for the assembly of cupA pilus.

Previous studies reported that Gac-associated hybrid histidine kinase, LadS, through a genuine phosphor-relay, which requires the histidine-phosphotransfer domain of GacS, activates Gac/Rsm cascade to facilitate the bacterial transition from a planktonic to a sessile lifestyle and promote biofilm formation (36, 37). Broder et al. further evidenced that calcium stimulates the Gac/Rsm cascade via the LadS that to facilitate the bacterial transition from acute-to-chronic switch (38). Here, we found that cupA pilus was regulated by LadS and essential for bacterial adhesion and the biofilm formation (Fig. 3F and G; Fig. S3BC). Considering the same final output from previous studies and our findings, one possible reason is that the cupA may be an early-stage effector for LadS, mediating the bacteria to a sessile growth. The LadS-Gac/Rsm cascade regulates the chronic infection switch and biofilm formation in a later stage. Collectively, the LadS is a vital sensor for bacterial adhesion. Mutations in *ladS*, without a normal phenotype of cupA pilus to transition from a planktonic to a sessile lifestyle, will result in an extended acute infection. The *P. aeruginosa* PA14 strain has a natural insertion mutation in the *ladS* gene, which differs from the PAO1 strain and eliminates its function in the histidine kinase domain (39, 40). Studies have shown that PAO1 strain quickly commits to a surface compared to PA14 strain (41).

How bacteria evade MΦs clearance during acute infections? Now, a plausible explanation is that, due to the ROS derived from MΦs, the expression of the LadS/PA0034 system was suppressed, after which the cupA pilus was downregulated, and the bacteria were not predisposed to a sessile growth but instead display an affinity for a free-living planktonic lifestyle. However, the mechanism by which fimbrial subunit cupA1 directly interacts with MΦs and mediates phagocytosis is a matter for further investigation. It is possible that the free-swimming state allows the bacteria diffusion and multiplication, making it difficult for MΦs to contain the infection, leading to more aggressive infections. This suggests a nuanced interaction where *P. aeruginosa* seemingly “recognizes” the threat posed by MΦs concealed behind ROS. As MΦs attempt to eliminate bacteria using ROS, the bacteria, in turn, alter their external appearance to escape MΦs, showcasing a complex and intelligent adaptive response.

## MATERIALS AND METHODS

### Bacteria culture

*P. aeruginosa* and *Escherichia coli* cells were routinely cultured in lysogeny broth (LB) at 37°C with shaking (200 rpm) unless otherwise specified. Acetamide plates were used exclusively for the growth of *P. aeruginosa*. Antibiotics were added to the medium as required: 50 µg/mL tetracycline or 400 µg/mL carbenicillin for *P. aeruginosa*; 100 µg/mL kanamycin or 100  µg/mL ampicillin for *E. coli*. The growth curves were monitored at OD_600_ by the Microscreen device (Gering, China) at intervals of 1 hour for a total of 21 hours (Fig. S2A).

For the macrophage uptake assay and mice acute lung infection assay, the bacteria were prepared as outlined below. Initially, the bacteria were cultured in LB with antibiotics overnight to achieve optimal growth. Subsequently, these cultures were transferred to fresh LB with an initial OD_600_ of 0.01 and were allowed to grow for 3 hours. Upon reaching the mid-log phase, as indicated by an OD_600_ of approximately 0.5, the bacteria were washed twice with phosphate buffered saline (PBS) , and their concentration was adjusted to an indicated value for subsequent experiments. For bacterial assay, overnight cultured bacteria were diluted in fresh LB medium to OD_600_ = 0.01 and cultured at 37°C at 200 rpm. The H_2_O_2_ was added directly to the LB medium at 10 µM, 50 µM, and 100 µM to treat the bacteria for a period of 3 hours.

### Cell culture

Murine macrophage-like RAW264.7 cells were cultured in RPMI-1640 medium supplemented with 10% fetal bovine serum at 37°C in a 5% CO_2_ environment. Inducing macrophage polarization was performed as previously (42). Briefly, untreated RAW264.7 cells were labeled as M0 macrophages. Cells were seeded in a 10 cm dish approaching 70% confluence. M1-like macrophages were obtained by stimulating RAW264.7 cells with lipopolysaccharide (100 ng/mL, Sigma-Aldrich). M2-like macrophages were obtained by treating RAW264.7 cells with IL-10 (50 ng/mL, BioLegend). After 24 hours of induction, the supernatants of culture media was collected by centrifuged at 2,000 × *g* for 10 minutes at 4°C to remove cell debris and immediately used for bacterial culture.

### Strains and plasmid

All strains and plasmids used in this study were described in Table S1. Mutant strains were constructed using the lambda red system as described previously (43). Successfully recombinant cells with positive colonies were further confirmed by PCR and DNA sequencing. The primers used in the construction of mutant strains are listed in Table S1. To create the compensatory plasmids for mutant strains, the alleles were ligated into pucp-Nde vector (linearized with EcoRI and HindIII) through a one-step cloning kit (10911ES20, YEASEN, China). The point mutations of *PA0034* allele (*D9N*, *D53N*, and *D9/53N*) were constructed with the Site-Directed Mutagenesis Kit (11003ES10, YEASEN, China). Appropriate antibiotics were used for the selection of ligation product as indicated in Table S1.

### Homolog identification and sequence analysis

Blastn searches were carried out on pseudomonas.com website to identify orthologs of 16S rDNA and *PA0034* across the *Pseudomonas* genus (Table S3). The 16S rDNA (PA4280.5) and *PA0034* sequence from the PAO1 strain served as the query sequences. Orthologs identified from the *Pseudomonas* genomes with E values of <1 × 10^−4^, percentage identity >90% and query coverage values of >90% were retained for subsequent maximum-likelihood tree analysis by MEGA11 and visualized in EVOLVIEW. Furthermore, the alignment of *PA0034* orthologs in *P. aeruginosa* popular strains was visualized in CLUSTALW.

### Immunoblot analysis

Bacteria were harvested by centrifuge and subsequently lysed in 1× SDS loading buffer at a ratio of 30 µL/mg bacteria. Western blotting was conducted through a standard SDS-PAGE as previously (44). The stripping and re-probing blot was conducted using antibody stripping buffer according to the instruction (Thermo Scientific, #46430). Briefly, after completing the phosphorylation detection, the blot was washed once for 5 minutes in Tris buffered saline (TBS) to remove the chemiluminescent substrate. Stripping buffer was added to cover the blot and incubated for 15 minutes at 37°C with gentle shaking. The stripping buffer was then removed, and the blot was washed twice for 5 minutes each in TBS with gentle shaking. To test for the removal of the primary antibody, the membrane was incubated with horseradish peroxidase-conjugated secondary antibody for 40–60 minutes, then washed twice for 5 minutes each in TBST with gentle shaking. The membrane was then incubated with the chemiluminescent substrate. If no signal was detected after 5 minutes of exposure, the primary antibody had been successfully removed from the antigen. After confirming that the membrane had been stripped properly, the second immunoprobing experiment could be performed. The membrane was blocked in 5% bovine serum albumin before proceeding with the second immunoprobing experiment. The specific primary antibodies including anti-PA0034 (1:500, prepared in our laboratory), anti-FLAG (1:1,000, #14793S, CST), anti-HA (1:1,000, #3724S, CST), anti-Rpoα (1:1,000, #663104, BioLegend), anti-*P38* (1:1,000, #9212S, CST), anti-p-*P38* (1:1,000, #9211S, CST), anti-JNK (1:1,000, #9252S, CST), anti-p-JNK (1:1,000, #4668S, CST), anti-ERK (1:1,000, #4695S, CST), and anti-p-ERK (1:1,000, #4370S, CST).

### RNA isolation and RT-qPCR

The RNA isolation was performed using TRIZOL reagent as previously (44). One milligram of total RNA was reverse transcribed to cDNA for further RT-qPCR analysis using SYBR Green. RT-qPCR was performed on an ABI 7500 RT-PCR system. The gene *rplu* served as endogenous control. Changes in gene expression were calculated using the ΔΔCt method, based on the mean change in RT-qPCR cycle threshold (ΔCt). Primers were designed using NCBI Primer-BLAST tool, and product melting curves were done to validate the specificity of the primers, with the specific sequences provided in Table S1.

### Co-immunoprecipitation

The co-immunoprecipitation was conducted as previously with a few modifications (45, 46). Briefly, the Flag tagged *PA0034* allele was expressed in Δ*PA0034* strain, using a strain carrying an empty vector as the control. Bacteria cultured in LB upon reaching the mid-log phase were harvested after twice PBS washing, and cells were lysed using lysozyme followed by ultrasonication lysis. The resultant lysates were combined with anti-Flag magnetic beads and incubated overnight at 4°C. Subsequent washing steps were carried out thrice using 1 mL PBST to eliminate non-specific proteins. The beads were then resuspended in 1× SDS loading buffer, boiled for 10 minutes, and subjected to SDS-PAGE. Protein bands were visualized via Coomassie blue staining, and the relevant gel bands were excised. The liquid chromatography tandem mass spectrometry analysis of the gel bands was conducted by Shanghai Applied Protein Technology Co., Ltd., as normal (47).

### The phosphotransfer assay

The phosphotransfer assay was conducted as previously with a few modifications (48). The Flag tagged *PA0034*, *PA0034(D9N*), *PA0034(D53N*), and *PA0034(D9/53N*) alleles were exogenously expressed in the Δ*PA0034* strain, and the HA-tagged *PA1458*, *PA3271*, *phoQ*, and *ladS* alleles were exogenously expressed in the PAO1 strain. Bacteria reaching the mid-log phase were harvested after twice PBS washing, and cells were lysed using lysozyme followed by ultrasonication lysis. For PA0034 and HKs phosphotransfer assay, the anti-Flag or anti-HA magnetic beads were added and incubated overnight at 4°C, and then magnetic beads were washed thrice using 1 mL PBST to eliminate non-specific proteins. The samples containing the Flag-PA0034 or HA-HKs were used for phosphotransfer assay. Magnetic beads containing HKs proteins were first auto-phosphorylated in kinase buffer (10 mM HEPES [pH7.3], 150 mM NaCl, and 10 mM MgCl_2_) by adding 1 mM ATPγS (ab138911, Abcam, USA) The volume was adjusted to 100 µL and spun at 30°C for 30 minutes. The beads containing PA0034 were replenished, and the reactions were run for another 30 minutes. Thiophosphates were alkylated by the addition of 2 mM p-nitrobenzyl mesylate (N274738, Aladdin, China) for 1 hour at room temperature. Adding 5× SDS loading buffer and bath in boiling water for 10 minutes. Samples were detected by SDS-PAGE using an anti-thiophosphate ester-specific antibody.

### Chromatin immunoprecipitation with q-PCR

The chromatin immunoprecipitation assay was conducted as previously with a few modifications (46). PAO1 strain exogenous expression of Flag tagged *PA0034* allele was used for the chromatin immunoprecipitation assay, and strain with empty vector was used as the control. One hundred milliliter culture reaching the mid-log phase was crosslinked using 1% formaldehyde at room temperature for 10 minutes. The cells were then pelleted in a pre-cooled centrifuge and washed twice in ice-cooled 1× TBS. Lysis of cells by lysozyme (5 mg/mL) at 37°C for 30 minutes, followed by sonication on ice (200W, duty cycle; total time, 4 minutes; time on, 8 s; time off, 12 s; 12 cycles). Magnetic beads with anti-Flag affinity were added to the cell lysate containing 25 µg of sheared DNA, followed by overnight incubation at 4°C. The magnetic beads–antigen–DNA complexes were washed to remove the non-specifically combination. The protein-DNA complexes were de-crosslinked by adding RNase A (1 mg/mL) and incubating at 65°C for 30 minutes, then Proteinase K was added (1 mg/mL) and incubated at 65°C for 2 hours. The DNA purification was performed using a DNA purification kit according to the manufacturer’s instructions (Qiagen, Germany). The purified input samples were run forward and used to verify fragment size and DNA concentration.

Primers targeting the cupA1 promoter regions for CHIP-qPCR were described in Table S1. Product melting curves were done to validate the specificity of the primers. Input and immunoprecipitated samples were detected by qPCR for fragment enrichment analysis. The ΔCt values of target fragments were normalized to their relative input sample, normalized ΔCt = Ct (CHIP) − [Ct (input) − Log2 (input dilution factor)]; enrichment of target fragments in immunoprecipitated samples was denoted as [% input], where % input = 2^−(normalized ΔCt)^ × 100%.

### RNA-seq analysis

RNA-seq analysis was performed by Guangdong Magigene Biotechnology Co., Ltd (Guangzhou, China) as normal. Briefly, the PAO1 and Δ*PA0034* strains were grown in LB with three biological replicates and collected at mid-log phase. Total RNA was extracted using standard procedures, and the RNA quantity was simultaneously measured using Qubit 3.0 (Thermo Fisher Scientific, MA, USA) and Nanodrop One (Thermo Fisher Scientific, MA, USA). The RNA integrity was accurately detected using the Agilent 4200 system (Agilent Technologies, Waldbron, Germany). Whole mRNAseq libraries were constructed by Guangdong Magigene Biotechnology Co. (Guangzhou, China) using the NEB Next Ultra Directional RNA Library Prep Kit for Illumina (New England Biolabs, MA, USA) in line with the manufacturer’s recommendations. The clustering of the index-coded samples was carried out on a cBot cluster generation system. After clustering, the library was sequenced on an Illumina Novaseq6000 platform, and 150 bp paired-end reads were generated. Differentially expressed genes were performed using DESeq2 (v1.34.0). FDR ≤ 0.05 and log2(fold change) ≥ 1 genes were classified as differentially expressed and were selected for further investigation. Significantly enriched GO terms were identified with an FDR threshold of ≤0.05.

### Macrophage uptake assay

Macrophage uptake assay was performed as previously with slight changes (49). Briefly, murine macrophage-like RAW264.7 cells were cultured in RPMI-1640 medium supplemented with 10% fetal bovine serum at 37°C in a 5% CO_2_ environment. RAW264.7 cells were seeded into 12-well plates at a concentration of 2.5 × 10^5^ cells/well cultured for 6 hours, and bacteria were added to start-up infection. For inhibition of the macrophage endocytosis, RAW264.7 cells were treated with 2 µM cytochalasin D (Aladdin) 30 minutes prior to infection as described previously (50). The bacterial solution was prepared as follows: *P. aeruginosa* grown in LB reached to mid-log phase (OD_600_ = 0.5) and adjusted to OD_600_ = 0.025 in RPMI-1640 medium after twice washing with PBS. The cell culture medium was then replaced with 1 mL bacterial solution per well, and the setup was incubated for 30 minutes at 37°C. Then, RAW264.7 cells were treated with 400 mg/mL gentamicin for 1 hour at 37°C to eliminate extracellular bacteria. After two PBS washes to remove residual bacteria, cells were lysed using 0.5% Triton X-100 for the stipulated time. For a preliminary assessment of bacterial numbers, cell lysates underwent a 10-fold dilution and were spot-like inoculated individually to predict bacterial loads via a pre-test. The suitably diluted lysate was spread on acetamide agar plates, and intracellular bacteria numbers were determined by CFU counting.

For confocal visualization of bacteria phagocytosis, RAW264.7 cells were seeded into the confocal dish (20 mm) and infected by bacteria as stated above. Subsequently, they were fixed with 4% paraformaldehyde for 1 hour, followed by cytoskeleton staining by phalloidin, *P. aeruginosa* staining by anti-PAO1 and FITC conjugated secondary antibodies, and nuclear staining using DAPI. Images were captured using a fluorescence confocal microscope (ZEISS 880), and the relative fluorescence intensity was analyzed through ImageJ 1.8.0.

For flow cytometry analysis of bacteria phagocytosis, the *P. aeruginosa* was pre-stained with SYTO9 live-cell dye according to the instructions and immediately infected with RAW264.7 cells for 30 minutes. The cells were washed twice with PBS, digested by trypsin, and fixed with 4% paraformaldehyde for 1 hour. Analysis was performed on a BD Fortesa flow cytometer, with data interpretation done using FlowJo 10.5.3 software.

### Mouse acute lung infection assay

The animal experiments were in accordance with the Army Medical University and Chinese national laboratory animal care and usage guidelines. To test the bacterial pathogenicity, the mouse acute lung infection model was conducted as previously with minor modifications (51). In brief, overnight cultured bacteria were re-inoculated into fresh LB medium with a starting concentration of OD_600_ = 0.01 and continued culturing for 3 hours so that the bacteria are in a log-phase (OD_600_ around 0.5). After sterile PBS washing, bacterial suspension was adjusted OD_600_ to 0.1, 0.3, and 0.5, and then 8-week-old male BALB/c mice were anesthetized by intraperitoneal injection of sodium pentobarbital (50 mg/kg) and then inoculated with 50 µL bacterial suspension via tracheotomy. The survival rate of the mice was monitored at 4-hour intervals, and the pathological sections of the lung 20 hours post infection were detected. Ultimately, the intermediate dose (OD_600_ = 0.3) that caused significant inflammatory infiltration and hemorrhage in the lungs of mice without killing them within 20 hours was selected for subsequent experiments. The survival rate of the mice was monitored at 4-hour intervals, spanning a total of 120 hours.

For pathological section staining and flow cytometry analysis of lung tissue, 20 hours post-infection, mice were sacrificed with CO_2_, and the lungs were removed for further use. For preliminary estimation of bacterial load of lung tissue, the tissue lysates underwent a 10-fold dilution and were spot-like inoculated individually to predict bacterial loads via a pre-test. The properly diluted lysates were plated on acetamide agar plates, and the bacterial load was determined by CFU counting.

### Flow cytometry

The flow cytometry analysis of lung tissue was conducted as previously with few modifications (52). Briefly, 20 hours post-infection, the right lobe of the lung was shred and digested in 1 mL digestive buffer (1 mg/mL collagenase D, 0.1 mg/mL DNase I, 2 mM L-glutamine, 2% FBS, and 25 mM HEPES resolved in Hanks' balanced salt solution) for 30 minutes at 37°C. Then the digested tissue was homogenized with syringe shanks prior to filtered through a 70 µm strainer. Erythrocytes present in the homogenates were lysed using a dedicated lysis buffer, followed by two washes. Any remaining red blood cells were lysed using ACK lysis buffer (GIBCO) for a duration of 2 minutes. Cells were washed with PBS and resuspended in PBS containing 1% FBS for further stained with fluorochrome-conjugated antibodies for 60 minutes at 4°C. Distinct leukocyte populations were characterized based on the following criteria: myeloid (CD45+CD11b+), neutrophils (CD45+CD11b+F4/80-Ly6G+), M1 MΦs (CD45+CD11b+F4/80+CD86+), and M2 MΦs (CD45+CD11b+F4/80+CD206+). Samples were assessed using a flow cytometer (BD Fortesa), and data were analyzed using FlowJo 10.5.3.

### Transmission electron microscopy

The cells and fimbriae were examined with a transmission electron microscope. The bacteria were inoculated in the liquid LB medium or solid LB plate as normal. Then bacterial suspension was performed and dropped on the formvar- and carbon-coated copper grids and negatively stained with 2% phosphotungstic acid. Electron microscopy pictures were taken with a TEM (JEM-1200EX; JEOL, Japan) operating at an accelerating voltage of 120 kV.

### Bacterial adhesion assay

The bacterial adhesion assay was performed as described previously (53) with minor changes. Briefly, polystyrene 24-well plates were pretreated with fetal bovine serum and air-dried for use. Overnight cultures of *P. aeruginosa* strains were diluted in LB medium to OD_600_ = 0.025, and 500 µL of bacterial suspensions were transferred into the pretreated 24-well plates. After a 3-hour incubation period at 37°C, the bacterial density in the culture medium was measured by optical density at 600 nm (OD_600_). The unattached cells on the bottom of the plate were removed by washing with PBS and air-dried. Subsequently, the volume of adherent bacteria was visualized using the standard gram stain.

### Crystal violet staining for biofilm formation

Crystal violet staining for biofilm formation assay was performed as previously described (54). Briefly, overnight cultures of *P. aeruginosa* strains were diluted in LB medium to OD_600_ = 0.025, and 500 µL of bacterial suspensions were transferred into the polystyrene 24-well plates. Biofilms were grown for 24 hours at 37°C with 40 rpm per minute. After that, the culture supernatant was discarded, cells were rinsed using phosphate buffered saline, and the attached biomass was stained with 0.5% crystal violet for 15 minutes. Subsequently, crystal violet was removed, biofilm-embedded cells were rinsed with PBS in order to remove unattached cells and residual crystal violet and then resuspended with 300 µL of 95% ethanol. After transfering the solution to a 96-well plate, the optical density of the released crystal violet was measured by means of the absorbance plate reader at 610 nm.

### Statistics and reproducibility

Statistical differences between group means were evaluated using a two-tailed Student’s *t* test or the Mann Whitney U test, based on the specific scenario. Statistical analyses were conducted using Prism GraphPad 8. A *P*-value < 0.05 was considered significant. The data points across the figures represent the mean ± SD of values as noted. Significance levels were denoted as: *P ≤ 0.05, **P ≤ 0.01, and ***P ≤ 0.001, with “ns” indicating not significant (*P* > 0.05). All experiments were replicated for reproducibility. The precise number (*n*) of samples in animal and *in vitro* studies was detailed in the figures and their legends.

## Data Availability

Data supporting the findings of this paper may be obtained from the corresponding author upon reasonable request. The RNA-seq data are uploaded to SRA with accession number PRJNA1026660.

## SUPPLEMENTAL MATERIAL

Figure S1 mbio.00616-24-s0001.tif Homology analysis of PA0034 in *P. aeruginosa* and other *Pseudomonas* species.

Figure S2 mbio.00616-24-s0002.tif *P. aeruginosa* PA0034 increased bacterial phagocytosis by MΦs.

Figure S3 mbio.00616-24-s0003.tif *P. aeruginosa* PA0034 increased bacterial adhesion.

Figure S4 mbio.00616-24-s0004.tif TEM imaging for the fimbrial structure on the outer membrane of *P. aeruginosa*.

Figure S5 mbio.00616-24-s0005.tif Fibrial protein cupA1 promoted bacterial phagocytosis by MΦs.

Figure S6 mbio.00616-24-s0006.tif *P. aeruginosa* LadS and PA0034 activation increased bacterial phagocytosis by MΦs.

Legends mbio.00616-24-s0007.docx Legends for supplemental material.

Table S1 mbio.00616-24-s0008.docx Strains, plasmids, and primers used in this study.

Table S2 mbio.00616-24-s0009.docx The RNA-seq analysis of pilus genes.

Table S3 mbio.00616-24-s0010.xls The analysis of PA0034 and PA0033 orthologs in the *Pseudomonas* genus.

ASM does not own the copyrights to Supplemental Material that may be linked to, or accessed through, an article. The authors have granted ASM a non-exclusive, world-wide license to publish the Supplemental Material files. Please contact the corresponding author directly for reuse.
