# Supplementary figures and images for: Pseudomonas aeruginosa two-component system LadS/PA0034 regulates macrophage phagocytosis via fimbrial protein cupA1
Source: mBio. 2024 May 21;15(6):e00616-24. doi: 10.1128/mbio.00616-24 (PMC11237798; doi:10.1128/mbio.00616-24)

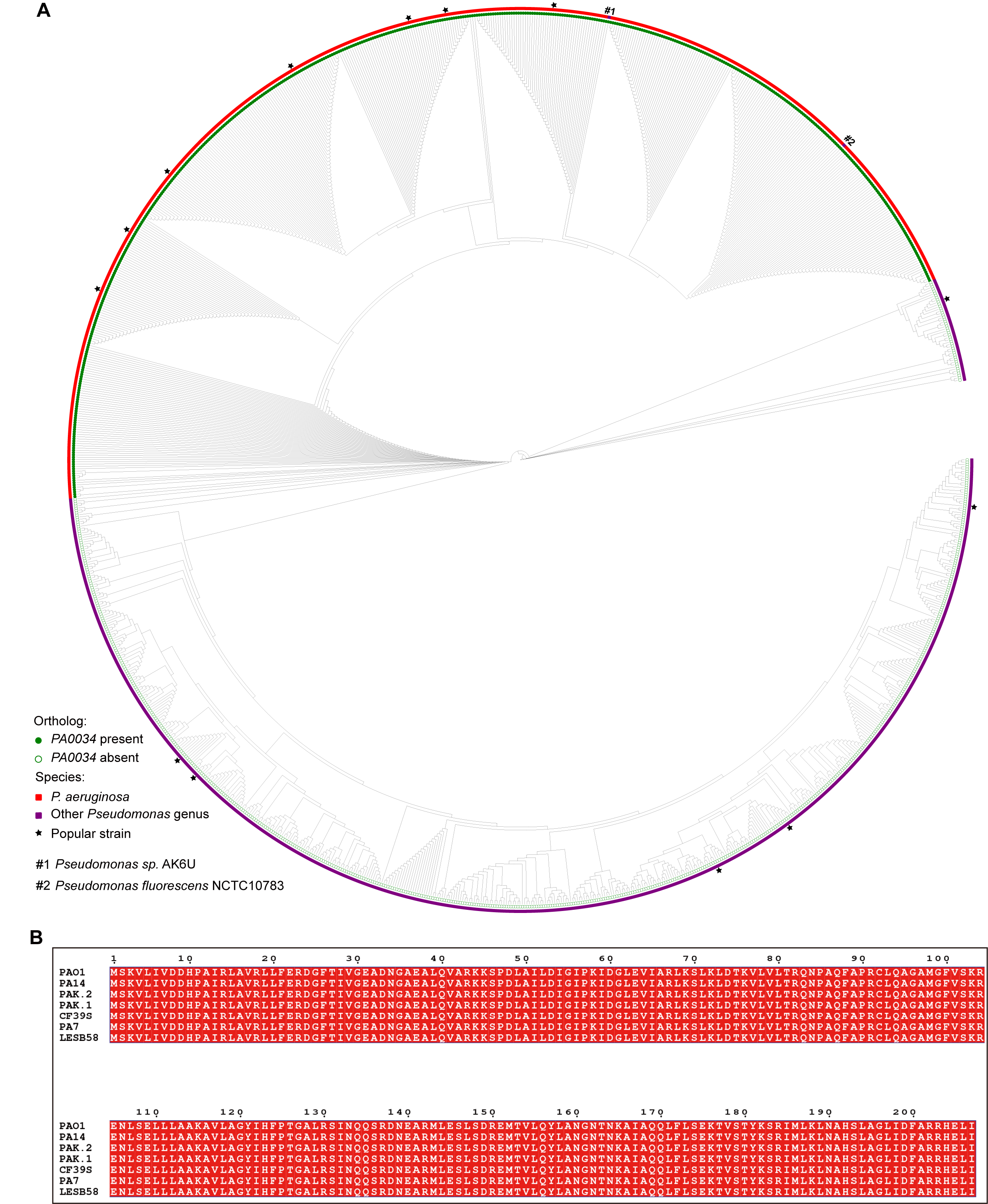

Supplement: Figure S1 — Homology analysis of PA0034 in P. aeruginosa and other Pseudomonas species. [file mbio.00616-24-s0001.tif]

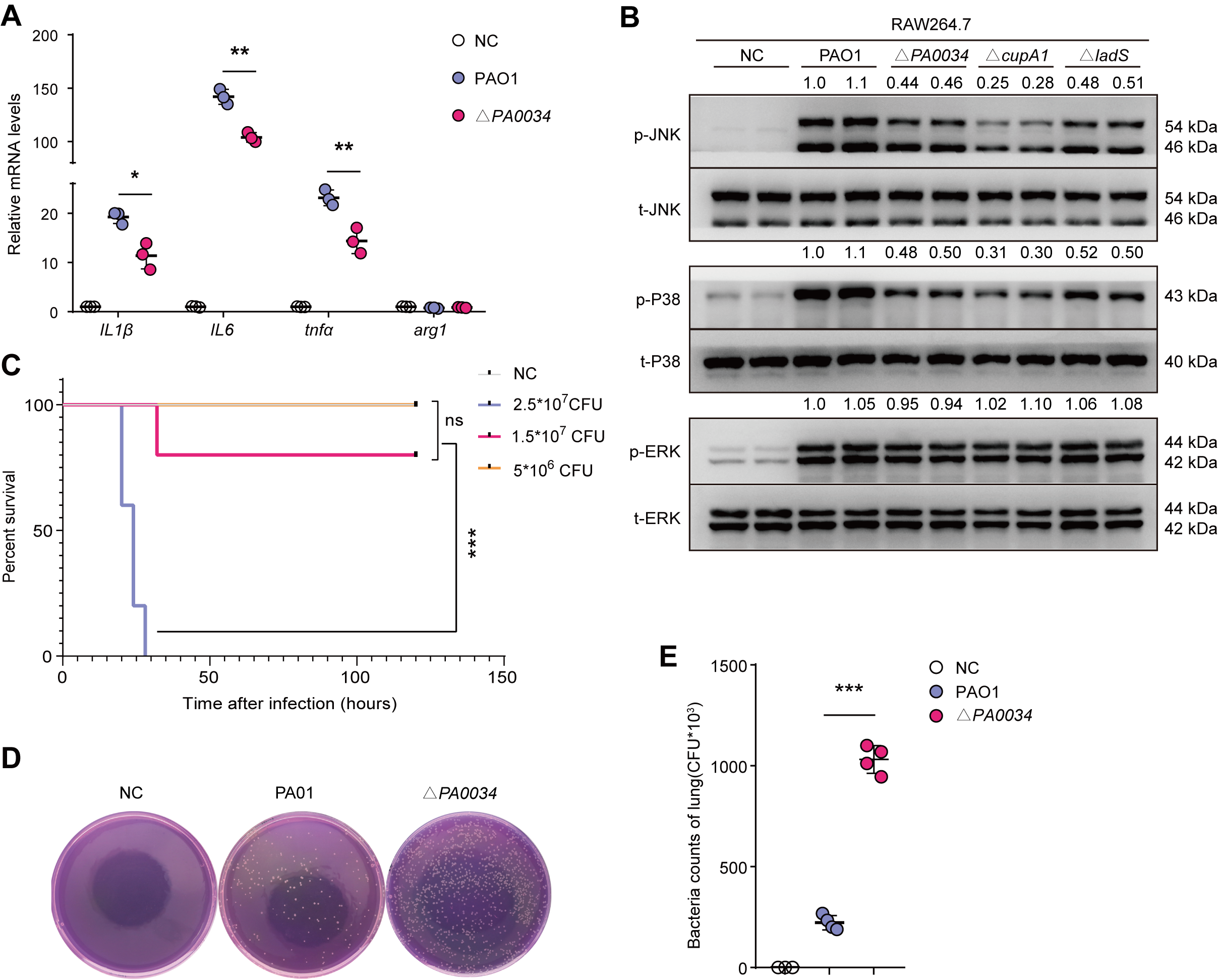

Supplement: Figure S2 — P. aeruginosa PA0034 increased bacterial phagocytosis by MΦs. [file mbio.00616-24-s0002.tif]

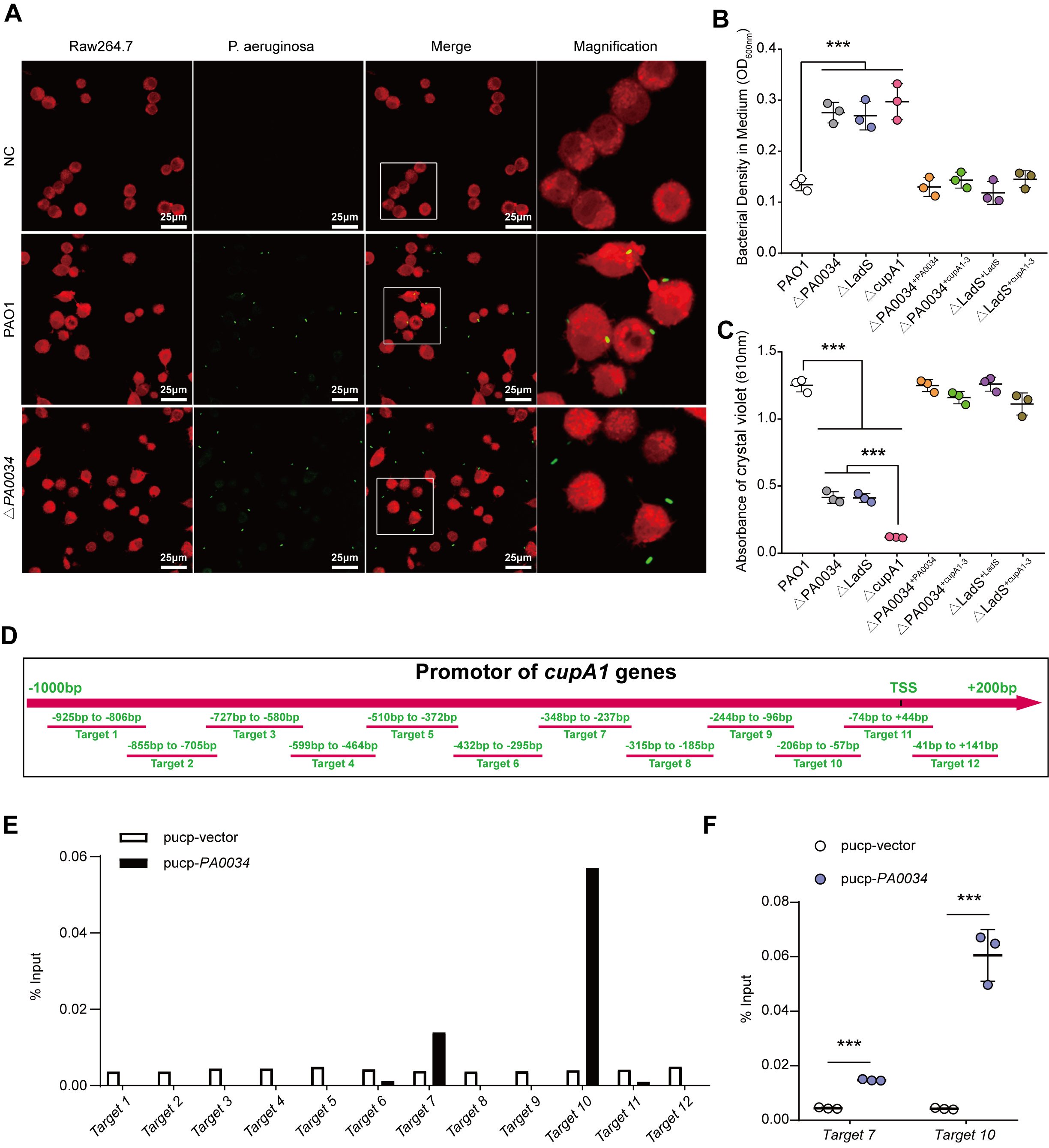

Supplement: Figure S3 — P. aeruginosa PA0034 increased bacterial adhesion. [file mbio.00616-24-s0003.tif]

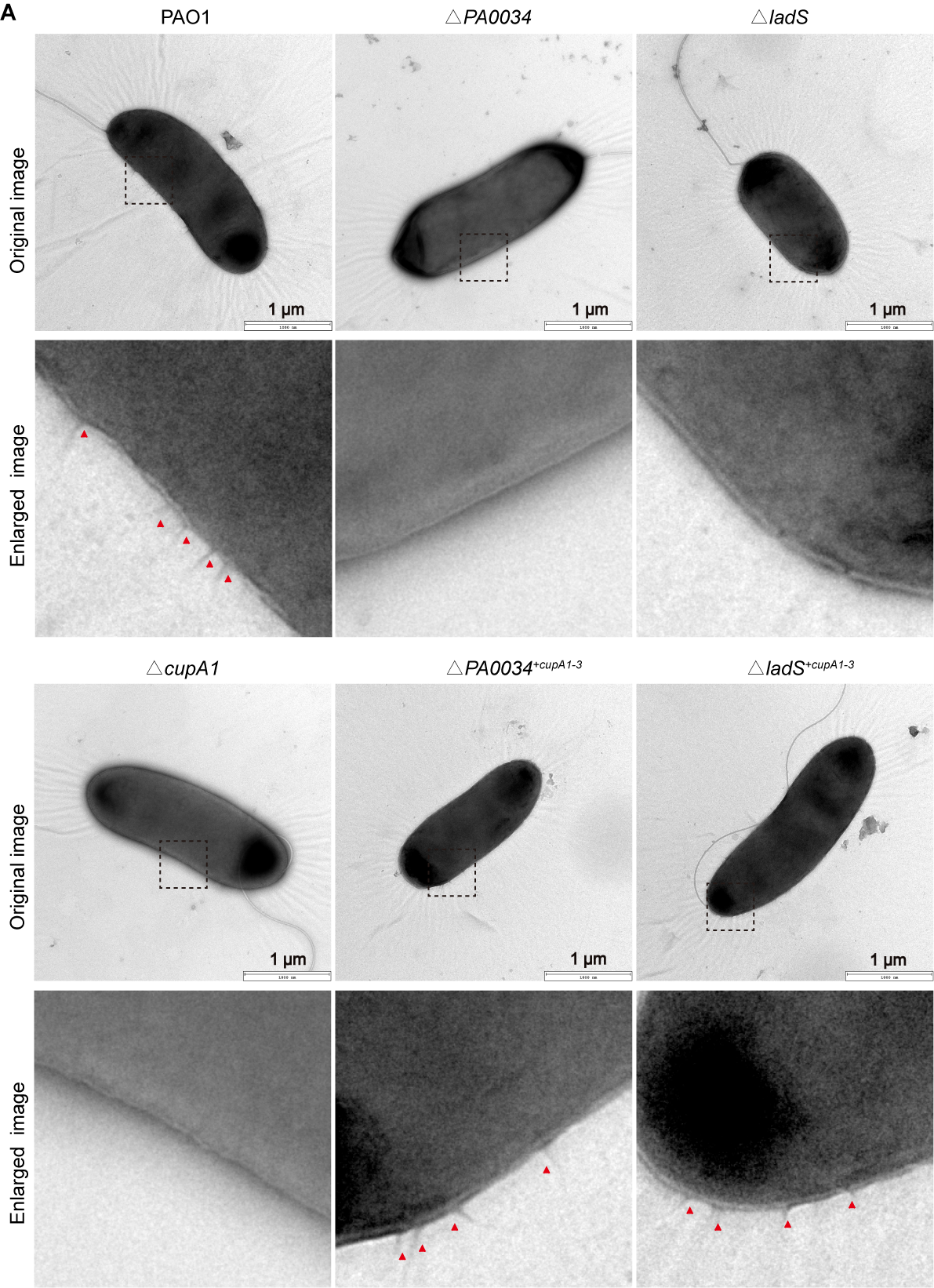

Supplement: Figure S4 — TEM imaging for the fimbrial structure on the outer membrane of P. aeruginosa. [file mbio.00616-24-s0004.tif]

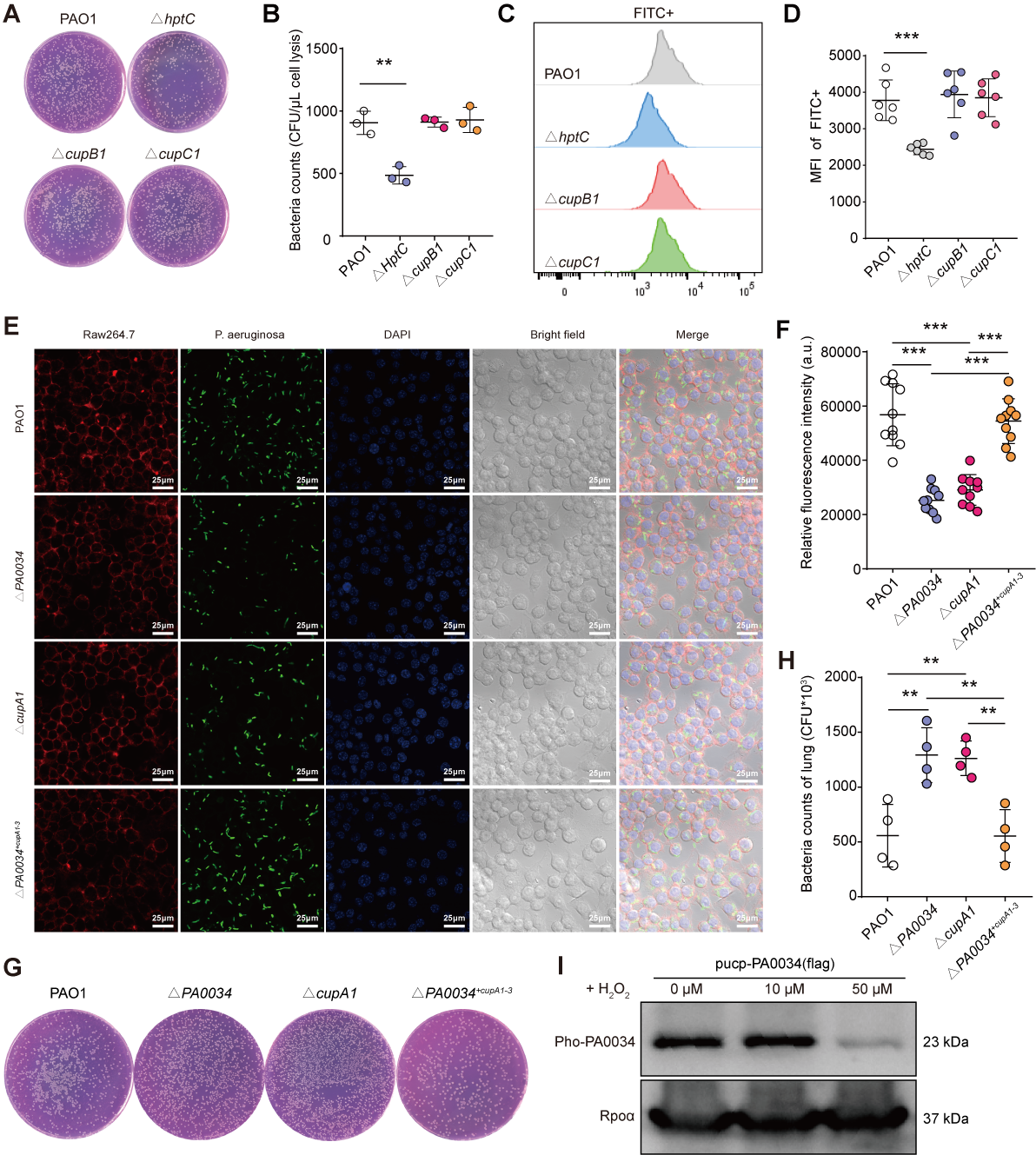

Supplement: Figure S5 — Fibrial protein cupA1 promoted bacterial phagocytosis by MΦs. [file mbio.00616-24-s0005.tif]

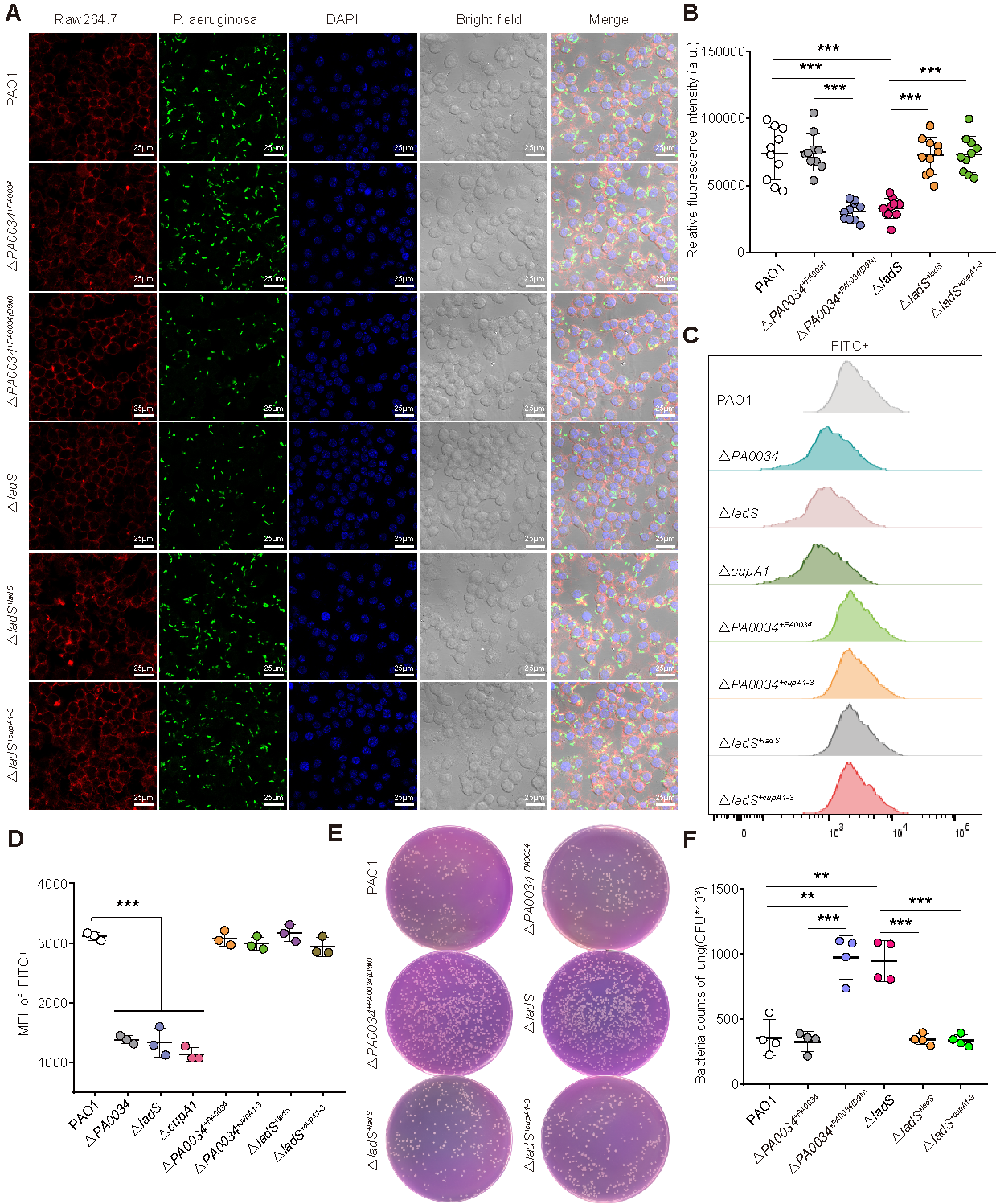

Supplement: Figure S6 — P. aeruginosa LadS and PA0034 activation increased bacterial phagocytosis by MΦs. [file mbio.00616-24-s0006.tif]
