# Supplementary material for: Pseudomonas aeruginosa two-component system LadS/PA0034 regulates macrophage phagocytosis via fimbrial protein cupA1
Source: mBio. 2024 May 21;15(6):e00616-24. doi: 10.1128/mbio.00616-24 (PMC11237798; doi:10.1128/mbio.00616-24)
Supplement: Legends — for supplemental material. [file mbio.00616-24-s0007.docx]

**Legends for Supplemental Figures and Tables**

**Section 1. Legends for Supplemental Figures**

**Figure S1** The homology analysis of *PA0034* in *P. aeruginosa* and other *Pseudomonas* genus. (**A**) The maximum-likelihood tree was built with 16s rDNA sequences across the *Pseudomonas* genus. *P. aeruginosa* species were indicated with red box, and the other *Pseudomonas* genus were indicated with violet box. The presence of the *PA0034* orthologues was indicated by a solid green circle, and the absence by a hollow circle. The star indicated the popular strains in *Pseudomonas*. (**B**) The protein sequences matching of PA0034 orthologues among all popular strains in *P. aeruginosa*.

**Figure S2** *P. aeruginosa* PA0034 increased the bacterial phagocytosis by MΦs.

(**A**) RT-qPCR analysis of *IL1β*, *IL6*, *tnf-α* and *arg1* mRNA levels in RAW264.7 cells after the infection of PAO1 and△*PA0034* strain, respectively. Saline-treated group was used as a normal control, (n=3). (**B**) The activation of MAPKs signaling pathways (ERK, JNK and P38) in RAW264.7 cells infected by PAO1, △*PA0034*, △*cupA1*, △*ladS* strains were detected by Western blotting through stripping and re-probing process. The intensity values of phosphorylated protein relative to total protein were marked upon. (**C**) 8-week male BALB/c mice were systemically infected with PAO1 strain via tracheotomy with 5*10^6^ CFU, 1.5*10^7^ CFU, 2.5*10^7^ CFU, respectively, Saline-treated group was used as a normal control. The survival of mice was monitored post the infection (n = 10). (**D**) The CFU counts of lung from acute lung infection mice induced by PAO1 and △*PA0034* strain were performed with the tissue lysates following serial dilution (n=4). (**E**) statistical analysis of CFU counts in figure 1D. The data shown were mean ±SD. Statistical significance by a two-tailed unpaired t-test is indicated: *P ≤ 0.05; **P ≤ 0.01; ***P ≤ 0.001; ns, not significant (P > 0.05).

**Figure S3** *P. aeruginosa* PA0034 increased the bacterial adhesion. (**A**) The live RAW264.7 cell was stained with phalloidin (red) and seeded in confocal dish at a concentration of 1*10^5^ cells per well for 4 hours, and then the cells were infected with live bacteria stained by SYTO9 dye (green). Confocal images were taken to represent the efficiency of bacterial capture by MΦs. Representative images from △PA0034 infected group compared to PAO1 were shown. (**B**) The bacterial density in culture medium from bacterial adhesive assay were measured by optical density at 600nm (n=3). (**C**) The crystal violet stained biofilm was quantified by absorbance measurement at 610 nm (n=3). (**D**) The schematic shows the specific primer sites that targets the *cupA1* promoter region. (**E**) The PA0034 was found to be enriched with two sites on the *cupA1* promoter (target 10 and target 7 site) by ChIP-qPCR. (**F**) The qPCR analysis of the CHIP samples on the target 10 and target 7 site specifically (n=3). The data shown were mean ±SD. Statistical significance by a two-tailed unpaired t-test is indicated: *P ≤ 0.05; **P ≤ 0.01; ***P ≤ 0.001; ns, not significant (P > 0.05).

**Figure S4** TEM imaging for the fimbrial structure on the outer membrane of *P. aeruginosa*. (**A**) The fimbrial structure of PAO1, △*PA0034*, △*ladS*, △*cupA1*, △*PA0034^+cupA1-3^*, and △*ladS^+cupA1-3^* strains cultured in solid LB medium were detected by transmission electron microscope (TEM), the scale of the original image on the upper level is 1μm. Long strips of pilus can be seen in all the strains at the original image. The small pilus on the outer membrane was displayed via enlarged scale below the original image as indicated by red arrow.

**Figure S5** Fimbrial protein cupA1 promoted the bacterial phagocytosis by MΦs. (**A** and **B**) The CFU counts and statistical analysis of bacteria engulfed by RAW264.7 cells in the PAO1, △*hptC*, △*cupB1*, and △*cupC1* strains infected groups (n=3). (**C** and **D**) FACS analysis for bacterial uptake by RAW264.7 cells in the PAO1, △*hptC*, △*cupB1*, and △*cupC1* strains infected groups. The histogram of FACS analysis and the mean fluorescence intensity (MFI) statistical graphs were shown (n=6). (**E** and **F**) Fluorescence confocal images showing the bacteria phagocyted by RAW264.7 cells in PAO1, △‍*PA0034*, △‍*cupA1* and △‍‍P*A0034^+cupA1-3^* infected groups. Representative images (E) and statistical analysis (F) were shown (n=3). The images were acquired from triplicate experiments, and analyzed by randomly selection of 10 fields of view. (**G** and **H**) Mice were infected with PAO1, △‍*PA0034*, △‍*cupA1* and △‍‍P*A0034^+cupA1-3^* strains with 1.5*10^7^CFU via tracheotomy for 20 hours. The CFU counts of lung were performed with the tissue lysates following serial dilution, and the statistical analysis were shown (n=4). (**I**) Western bloting for the phosphorylation of PA0034 in △‍‍*PA0034^+PA0034^* strain treated with H_2_O_2_. The pucp-PA0034(flag) was expressed in △‍‍*PA0034* strain and purified by anti-flag magnetic bead. The data shown were mean ±SD. Statistical significance by a two-tailed unpaired t-test is indicated: *P ≤ 0.05; **P ≤ 0.01; ***P ≤ 0.001.

**Figure S6** *P. aeruginosa* LadS and PA0034 activation increased the bacterial phagocytosis by MΦs. (**A**) Representative confocal images showing the bacteria phagocyted by RAW264.7 cells in PAO1, △*‍‍PA0034^+PA0034^*, △‍‍*PA0034^+PA0034(D9N)^*, △*‍ladS*, △‍‍*ladS^+ladS^* and △‍*ladS^+cupA1-3^* infected groups. And (**B**) statistical analysis were shown. The images were acquired from triplicate experiments, and analyzed by randomly selection of 10 fields of view. (**C** and **D**) RAW264.7 cells were pre-incubated with the actin cytoskeletal depolymerizing agent cytD to inhibit macrophage endocytosis. FACS analysis for bacteria captured by RAW264.7 cells in PAO1, △*PA0034*, △*cupA1*, △*‍ladS*, △*‍‍PA0034^+PA0034^*, △‍‍*PA0034^+cupA1-3^*, △‍‍*ladS^+ladS^* and △‍*ladS^+cupA1-3^* infected groups, and the mean fluorescence intensity (MFI) statistical graphs were shown. (**E** and **F**) Mice were infected with PAO1, △*‍‍PA0034^+PA0034^*, △‍‍*PA0034^+PA0034(D9N)^*, △*‍ladS*, △‍‍*ladS^+ladS^* and △‍*ladS^+cupA1-3^* strains with 1.5*10^7^CFU via tracheotomy for 20 hours. The CFU counts of lung were performed with the tissue lysates following serial dilution, and the statistical analysis were shown (n=4). The data shown were mean ±SD. Statistical significance by a two-tailed unpaired t-test is indicated: *P ≤ 0.05; **P ≤ 0.01; ***P ≤ 0.001.

**Section 2. Legends for Supplemental Tables**

**Table S1.** The Strains, plasmids and primers used in this study.

**Part 1.** The different genotype of *P. aeruginosa* strains used in this study, and the competent cells of *E. coli* strains used for gene expression and plasmid amplification. **Part 2.** The list of plasmids constructed in this study, and the description of each vector was indicated in the table. **Part 3.** The list of primers used in this study, and the purpose of each primer was indicated in the table.

**Table S2.** The reduced expression of pilus genes in △*PA0034* compared to PAO1 strains, data from RNA-seq analysis.

**Table S3.**

**Part 1.** The sequences of 16s rDNA orthologs analysis in *Pseudomonas* genus, including the P. aeruginosa and other *Pseudomonas* genus. **Part 2.** The sequences of *PA0034* orthologs analysis in *Pseudomonas* genus, including the P. aeruginosa and other *Pseudomonas* genus. **Part 3.** The sequences of *PA0033* (*hptc*) orthologs analysis in *Pseudomonas sp. AK6U* and *Pseudomonas fluorescens NCTC10783* strains.
