# Supplementary material for: Pseudomonas aeruginosa two-component system LadS/PA0034 regulates macrophage phagocytosis via fimbrial protein cupA1
Source: mBio. 2024 May 21;15(6):e00616-24. doi: 10.1128/mbio.00616-24 (PMC11237798; doi:10.1128/mbio.00616-24)
Supplement: Table S1 — Strains, plasmids, and primers used in this study. [file mbio.00616-24-s0008.docx]

| **Strain name** | **Description** | **Source** |
| --- | --- | --- |
| P. aeruginosa PAO1 | Wild-type laboratory strain. | Laboratory preservation |
| PAO1 (λ-Red) | PAO1 transformed with a λ-Red recombination system; Cb^R^. | Laboratory preservation |
| Δ*PA0034* | Deletion of PA0034 in PAO1; Tet^R^, Cb^R^. | This study |
| Δ*cupA1* | Deletion of cupA1 in PAO1; Tet^R^, Cb^R^. | This study |
| Δ*fimU-X* | Continuously deletion of *fimU*, *pilV*, *pilW* and *pilX* in PAO1; Tet^R^, Cb^R^. | This study |
| Δ*pilY1-2* | Continuously deletion of *pilY1* and *pilY2* in PAO1; Tet^R^, Cb^R^. | This study |
| Δ*pilE* | Deletion of pilE in PAO1; Tet^R^, Cb^R^. | This study |
| Δ*LadS* | Deletion of LadS in PAO1; Tet^R^, Cb^R^. | This study |
| Δ*hptC* | Deletion of hptC in PAO1; Tet^R^, Cb^R^. | This study |
| Δ*cupB1* | Deletion of cupB1 in PAO1; Tet^R^, Cb^R^. | This study |
| Δ*cupC1* | Deletion of cupC1 in PAO1; Tet^R^, Cb^R^. | This study |
| PAO1*^+LadS^* | PAO1 complemented with a HA tagged *ladS* allele (pucp-Nde plasmid-based expression); Cb^R^. | This study |
| PAO1*^+PA1458^* | PAO1 complemented with a HA tagged *PA1458* allele (pucp-Nde plasmid-based expression); Cb^R^. | This study |
| PAO1*^+PA3271^* | PAO1 complemented with a HA tagged *PA3271* allele (pucp-Nde plasmid-based expression); Cb^R^. | This study |
| PAO1*^+phoQ^* | PAO1 complemented with a HA tagged *phoQ* allele (pucp-Nde plasmid-based expression); Cb^R^. | This study |
| Δ*PA0034^+PA0034^* | Δ*PA0034* complemented with a Flag tagged *PA0034* allele (pucp-Nde plasmid-based expression). | This study |
| Δ*PA0034^+PA0034(D9N)^* | Δ*PA0034* complemented with a D9N site mutant *PA0034* allele (pucp-Nde plasmid-based expression). | This study |
| Δ*PA0034^+PA0034(D53N)^* | Δ*PA0034* complemented with a D53N site mutant *PA0034* allele (pucp-Nde plasmid-based expression). | This study |
| Δ*PA0034^+PA0034(D9/53N)^* | Δ*PA0034* complemented with D9N and D53N double sites mutant *PA0034* allele(pucp-Nde plasmid-based expression). | This study |
| Δ*PA0034^+cupA1-3^* | Δ*PA0034* complemented with a continuously *cupA1*, *cupA2* and *cupA3* allele (pucp-Nde plasmid-based expression). | This study |
| Δ*ladS^+ladS^* | Δ*ladS* complemented with a HA tagged *ladS* allele (pucp-Nde plasmid-based expression). | This study |
| Δ*ladS^+cupA1-3^* | Δ*ladS* complemented with a continuously *cupA1*, *cupA2* and *cupA3* allele (pucp-Nde plasmid-based expression). | This study |
| BL21 (DE3) | E. coli gene expression strain. | Laboratory preservation |
| DH5α | E. coli cloning strain. | Laboratory preservation |

Extended data Table 1. Strains, plasmids and primers used in this study

Part 1. The list of different strains used in this study

Part 2. The list of plasmids used in this study

| **Plasmid name** | **Description** | **Source** |
| --- | --- | --- |
| pucp-Nde | Parental plasmid used for gene expression in P. aeruginosa; Cb^R^ | Laboratory preservation |
| pucp-*PA0034* | The pucp plasmid carrying a Flag tagged *PA0034* allele; Cb^R^ | This study |
| pucp-*PA0034(D9N)* | The pucp plasmid carrying a Flag tagged *PA0034* allele harboring a mutant site (the aspartate residue 9 was substituted by asparagine residue); Cb^R^ | This study |
| pucp-*PA0034(D53N)* | The pucp plasmid carrying a Flag tagged *PA0034* allele harboring a mutant site (the aspartate residue 53 was substituted by asparagine residue); Cb^R^ | This study |
| pucp-*PA0034(D9/53N)* | The pucp plasmid carrying a Flag tagged *PA0034* allele harboring two mutant sites (the aspartate residue 9 and 53 was substituted by asparagine residue); Cb^R^ | This study |
| pucp-*ladS* | The pucp plasmid carrying a HA tagged *ladS* allele; Cb^R^ | This study |
| pucp-*PA1458* | The pucp plasmid carrying a HA tagged *PA1458* allele; Cb^R^ | This study |
| pucp-*PA3271* | The pucp plasmid carrying a HA tagged *PA3271* allele; Cb^R^ | This study |
| pucp-*phoQ* | The pucp plasmid carrying a HA tagged *phoQ* allele; Cb^R^ | This study |
| pucp-*cupA1-3* | The pucp plasmid carrying *cupA1*, *cupA2* and *cupA3* allele continuously; Cb^R^ | This study |

Part 3. The list of primers used in this study

| **Primer names** | **Sequence (from 5’ to 3’)** | **Purpose** |
| --- | --- | --- |
| PAO1 *rplU*-FP | CGCAGTGATTGTTACCGGTG | RT-PCR |
| PAO1 *rplU*-RP | GGTAACCTTCGCACCTTCGA | RT-PCR |
| PAO1 *cupA1* FP | TGGCGGCAAACACTATCACA | RT-PCR |
| PAO1 *cupA1* RP | AATCGGTGAGTTGCAGGGTG | RT-PCR |
| PAO1 *cupA2* FP | ATGCCCAGACATTCGTACCG | RT-PCR |
| PAO1 *cupA2* RP | TCAGTTGCACGGTCTTCTCC | RT-PCR |
| PAO1 *cupA3* FP | GATTCCGGCAGCTACAAGCA | RT-PCR |
| PAO1 *cupA3* RP | TGCCCCCAGGTATTGGAGTA | RT-PCR |
| PAO1 *cupA4* FP | GCCCTATTCCTACACCCAGC | RT-PCR |
| PAO1 *cupA4* RP | AGCGTTTCAGGGTCACGTAG | RT-PCR |
| PAO1 *cupA5* FP | GAATCGCTGTACTGGCTGAAC | RT-PCR |
| PAO1 *cupA5* RP | GTACAGCACCTTCATCTGGGT | RT-PCR |
| PAO1 *PA2133* FP | GATGGATGCCTGGTGGGAAG | RT-PCR |
| PAO1 *PA2133* RP | GAAGTCCAGGTTGTTGTGCG | RT-PCR |
| PAO1 *cupB1* FP | GGTAGCGAATACGGGACAGG | RT-PCR |
| PAO1 *cupB1* RP | ACCAACACCACGAAGATCCC | RT-PCR |
| PAO1 *cupC1* FP | TGACCACCGTGAAGCTGAAG | RT-PCR |
| PAO1 *cupC1* RP | CTTGCCATCGGAGCCATAGA | RT-PCR |
| PAO1 *pilE* FP | ACGTTGCTGGAAATGGTGGT | RT-PCR |
| PAO1 *pilE* RP | GGTTCTGCGAGTAGTAGCGT | RT-PCR |
| PAO1 *pilY2* FP | CTCCGTGGTGTCCTACTCTG | RT-PCR |
| PAO1 *pilY2* RP | GGGGCTGTTCTTTCTCGATCA | RT-PCR |
| PAO1 *fimU* FP | CGCCATTCCGAACTTCAAGC | RT-PCR |
| PAO1 *fimU* RP | GCCAGGACACCTATGCTCAG | RT-PCR |
| PAO1 *pilY1* FP | CGCAGCGGGAGTATCAATCT | RT-PCR |
| PAO1 *pilY1* RP | GTTCTGCCTTTGCTCTGTGC | RT-PCR |
| PAO1 *pilV* FP | AGCGACTACTACATCTGCCG | RT-PCR |
| PAO1 *pilV* RP | TGAGGGTGTAGTAGCAGAGGG | RT-PCR |
| PAO1 *pilX* FP | AGTGGAACAACCTGCCCATT | RT-PCR |
| PAO1 *pilX* RP | GGTTTCGCTGTTGGTCTGGT | RT-PCR |
| PAO1 *pilW* FP | AGCAAGACCCTGGACGATTG | RT-PCR |
| PAO1 *pilW* RP | ATTCCTGAGTGTCTGGCTGC | RT-PCR |
| PAO1 *ladS* FP: | AACCGATCAGGAATGGCGTG | RT-PCR |
| PAO1 *ladS* RP: | CTTCGTTCGGGCTCTACCAG | RT-PCR |
| PAO1 *PA0034* FP: | ATCAACCAGCAGAGCCG | RT-PCR |
| PAO1 *PA0034* RP: | TGGCCTTGTTGGTATTGC | RT-PCR |
| PAO1 *PA0179* FP | GCGTCACTTCGAGCAGTTTC | RT-PCR |
| PAO1 *PA0179* RP | CCTCATGCTGACCACCGAAT | RT-PCR |
| PAO1 *PA0756* FP | TGATGAGCAAGGAGCAACTGG | RT-PCR |
| PAO1 *PA0756* RP | GGTGGACGTAGATCTCGATGG | RT-PCR |
| PAO1 *PA1157* FP | TGACCCGGGAATACCTGGAA | RT-PCR |
| PAO1 *PA1157* RP | GTGAGCATCAGGATCGGACC | RT-PCR |
| PAO1 *PA1397* FP | CAGGGCAAGAGCAACAAGGA | RT-PCR |
| PAO1 *PA1397* RP | AGCAGCGACGACACATGGAT | RT-PCR |
| PAO1 *PA1437* FP | GACACCAATGTCGTGGAGGT | RT-PCR |
| PAO1 *PA1437* RP | AGCTTGTTCTCGAACGGACC | RT-PCR |
| PAO1 *PA2572* FP | TCGGACCTGCTCTATCACCA | RT-PCR |
| PAO1 *PA2572* RP | CTTGAGGTGGTCCGGATAGC | RT-PCR |
| PAO1 *PA2798* FP | CATCGAGGATGCCATCGGAA | RT-PCR |
| PAO1 *PA2798* RP | ACGATGCGACCTATGACGAC | RT-PCR |
| PAO1 *PA2881* FP | TCGGTGAGCACCTCGTAATG | RT-PCR |
| PAO1 *PA2881* RP | CTCCCGTCCGCATTCTCTTC | RT-PCR |
| PAO1 *PA3714* FP | GAAAACACGCGTCATCCTCG | RT-PCR |
| PAO1 *PA3714* RP | GTCGTACGCACTCAGCAGAT | RT-PCR |
| PAO1 *PA4032* FP | TCCGTAGCCGTAGATGGTCA | RT-PCR |
| PAO1 *PA4032* RP | CCGTTGTCGCGGGAATATCT | RT-PCR |
| PAO1 *PA5364* FP | TCAACAGCAGCCAATCGCTA | RT-PCR |
| PAO1 *PA5364* RP | TGGACCTCGAGGAAAACAGC | RT-PCR |
| Preimer 1 (-925) FP: | TCCGGAATACCAGTCGGAGG | CHIP-qPCR |
| Preimer 1 (-806) RP: | CGCAACGGTCTCCCTCAATA | CHIP-qPCR |
| Preimer 2 (-855) FP: | CATGGGTGCGAACCTCTTCT | CHIP-qPCR |
| Preimer 2 (-705) RP: | ACTGATTCGGAGTCCAACGTC | CHIP-qPCR |
| Preimer 3 (-727) FP: | CGACGTTGGACTCCGAATCA | CHIP-qPCR |
| Preimer 3 (-580) RP: | CCGTCTTGCGACAAGTCAAC | CHIP-qPCR |
| Preimer4 (-599) FP: | TTGACTTGTCGCAAGACGGA | CHIP-qPCR |
| Preimer4 (-464) RP: | CTTGCGCAGCAAGCTTTTTC | CHIP-qPCR |
| Preimer5 (-510) FP: | GCGCACCGGACTTCTGAGC | CHIP-qPCR |
| Preimer5 (-372) RP: | CCAAGTTGGAGTATGCACTT | CHIP-qPCR |
| Preimer6 (-432) FP: | CCGGGAAAAGTCGACGGTAA | CHIP-qPCR |
| Preimer6 (-295) RP: | GCTTGTCTTCGCCAGTTTGG | CHIP-qPCR |
| Preimer7 (-348) FP: | TGCATTACACGGATGACGGT | CHIP-qPCR |
| Preimer7 (-237) RP: | GTCGACATTGCAACGCTTGT | CHIP-qPCR |
| Preimer8 (-315) FP: | CCAAACTGGCGAAGACAAGC | CHIP-qPCR |
| Preimer8 (-185) RP: | ATTCGGGGAATTGCCTCGTT | CHIP-qPCR |
| Preimer9 (-244) FP: | TGTCGACGGATAGCACGAAG | CHIP-qPCR |
| Preimer9 (-96) RP: | TGGTGCGAACCACCGATATAA | CHIP-qPCR |
| Preimer10 (-206) FP: | AAACGAGGCAATTCCCCGAA | CHIP-qPCR |
| Preimer10 (-57) RP: | ACGCGGTAAAGACTCGAACG | CHIP-qPCR |
| Preimer11 (-74) FP: | TCGAGTCTTTACCGCGTTCC | CHIP-qPCR |
| Preimer11 (+44) RP: | GCAAAGGCCAATACCACTGC | CHIP-qPCR |
| Preimer12 (-41) FP: | AGGCGGGTGGACGATTATTG | CHIP-qPCR |
| Preimer12 (+104) RP: | ACTTCGCCGCTGAATGTGAT | CHIP-qPCR |

| pucp-*PA0034*-FP | TATAAAGCTTATGAGTAAGGTGCTGATCGTCG | pucp-*PA0034*-Flag cloning |
| --- | --- | --- |
| pucp-*PA0034*-RP | TATAGAATTCTCACTTATCGTCGTCATCCTTGTAATCGATCAGCTCGTGGCGGCGGG | pucp-*PA0034*-Flag cloning |
| pucp-*cupA1-3*-FP | GACCATGATTACGCCATGTACCCATACGATGTTCCAGATTACGCTACCAGAACTTCGAAC | pucp-HA-*cupA1-3* cloning |
| pucp-*cupA1-3*-RP | GAGCTCGGTACCCGGTCATCTGGCGTTCTCCA | pucp-HA-*cupA1-3* cloning |
| pucp-*PA1458*-FP | GACCATGATTACGCCATGAGCTTCGACGCC | pucp-*PA1458*-HA cloning |
| pucp-*PA1458*-RP | GAGCTCGGTACCCGGTCAAGCGTAATCTGGAACATCGTATGGGTAGATGCGCCGTGCGTA | pucp-*PA1458*-HA cloning |
| pucp-*PA3271*-FP | GACCATGATTACGCCATGTCGCTGTCCATCGG | pucp-*PA3271*-HA cloning |
| pucp-*PA3271*-RP | GAGCTCGGTACCCGGTCAAGCGTAATCTGGAACATCGTATGGGTAGCGCAGGCTCAGATG | pucp-*PA3271*-HA cloning |
| pucp-*phoQ*-FP | GACCATGATTACGCCGTGATCCGTTCCCTG | pucp-*phoQ*-HA cloning |
| pucp-*phoQ*-RP | GAGCTCGGTACCCGGTCAAGCGTAATCTGGAACATCGTATGGGTAGACTGTAGCGAAACG | pucp-*phoQ*-HA cloning |
| pucp*-ladS*-FP | GACCATGATTACGCCATGCGGCACTGGCTGAT | pucp-*ladS*-HA cloning |
| pucp-*ladS*-RP | GAGCTCGGTACCCGGTCAAGCGTAATCTGGAACATCGTATGGGTAGGCGGACTTGGTGAC | pucp-*ladS*-HA cloning |
| *PA0034-D9N*-FP | AATCATCCCGCTATCCGCCTGGCCGTGCGCTTGCTGTTC | Pucp-*PA0034-D9N*  mutagenesis |
| *PA0034-D9N*-RP | CAGGCGGATAGCGGGATGATTATCGACGATCAGCACCTTACTCA | Pucp-*PA0034-D9N*  mutagenesis |
| *PA0034-D53N*-FP | AACATCGGCATCCCCAAGATCGACGGTCTCGAAGTGATAGCCC | Pucp-*PA0034-D53N*  mutagenesis |
| *PA0034-D53N*-RP | GATCTTGGGGATGCCGATGTTCAGGATGGCCAGGTCGGGA | Pucp-*PA0034-D53N*  mutagenesis |
| *PA0034*-FA-FP | CTGCCGATCGGTATCGGCT | *PA0034* deletion |
| *PA0034*-FA-RP | AAGCTGTCAAACATGAGAATCATGCGAACCTTCTTTGAATG | *PA0034* deletion |
| *PA0034*-Tet^R^-FP | CATTCAAAGAAGGTTCGCATGATTCTCATGTTTGACAGCTT | *PA0034* deletion |
| *PA0034*-Tet^R^-RP | CAGGTCGAACGTTCGCATGTCAGGTCGAGGTGGCCCG | *PA0034* deletion |
| *PA0034*-BA-FP | CGGGCCACCTCGACCTGACATGCGAACGTTCGACCTG | *PA0034* deletion |
| *PA0034*-BA-RP | TCATCCAGTCGTGCAGTTCG | *PA0034* deletion |
| *ladS*-FA-FP | CGCTGACCATGAGCTACGCCG | *ladS* deletion |
| *ladS*-FA-RP | CGTGCCTTCATCCGTTTCCGCAGTTCGTGGGTGACGGTGG | *ladS* deletion |
| *ladS*-Tet^R^-FP | CCACCGTCACCCACGAACTGCATGATTCTCATGTTTGACAGCTT | *ladS* deletion |
| *ladS*-Tet^R^-RP | CGTTGTCCTCCACCACCAGCATCAGGTCGAGGTGGCCCG | *ladS* deletion |
| *ladS*-BA-FP | ACCCAAGTACCGCCACCTAATGCTGGTGGTGGAGGACAACG | *ladS* deletion |
| *ladS*-BA-RP | TCAGGCGGACTTGGTGACGAT | *ladS* deletion |
| *cupA1*-FA-FP | TTCGAGTCTTTACCGCGTTCC | *cupA1* deletion |
| *cupA1*-FA-RP | CGTGCCTTCATCCGTTTCCGCCTATGTTCCTTGTGTGA | *cupA1* deletion |
| *cupA1*-Tet^R^-FP | TCACACAAGGAACATAGGCATGATTCTCATGTTTGACAGCTT | *cupA1* deletion |
| *cupA1*-Tet^R^-RP | CGCATGCGCCGCCGGCATTCAGGTCGAGGTGGCCCG | *cupA1* deletion |
| *cupA1*-BA-FP | ACCCAAGTACCGCCACCTAAATGCCGGCGGCGCATGCG | *cupA1* deletion |
| *cupA1*-BA-RP | CGGTACGAATGTCTGGGCATG | *cupA1* deletion |
| *fimU-pilV/WX*-FA-FP | AAGACGGTGGGGAACGGCTT | *fimU-pilV/WX* deletion |
| *fimU-pilV/WX*-FA-RP | CGTGCCTTCATCCGTTTCCGCGGTTGCTCCAGGGGGTCTA | *fimU-pilV/WX* deletion |
| *fimU-pilV/WX*-Tet^R^-FP | TAGACCCCCTGGAGCAACCGCATGATTCTCATGTTTGACAGCTT | *fimU-pilV/WX* deletion |
| *fimU-pilV/WX*-Tet^R^-RP | GATTTCATGCGAGGCTCGATCAGGTCGAGGTGGCCCG | *fimU-pilV/WX* deletion |
| *fimU-pilV/WX*-BA-FP | ACCCAAGTACCGCCACCTAATCGAGCCTCGCATGAAATC | *fimU-pilV/WX* deletion |
| *fimU-pilV/WX*-BA-RP | CACCAGTGTGAGTTTCTTCGG | *fimU-pilV/WX* deletion |
| *pilY1-2*-FA-FP | GCAACCAGACCAACAGCGAA | *pilY1-2* deletion |
| *pilY1-2*-FA-RP | CGTGCCTTCATCCGTTTCCGCGAGGCTCGATCAGTTGGTA | *pilY1-2* deletion |
| *pilY1-2*-Tet^R^-FP | TACCAACTGATCGAGCCTCGCATGATTCTCATGTTTGACAGCTT | *pilY1-2* deletion |
| *pilY1-2*-Tet^R^-RP | TTCTGTCTTGTCCTCATCGGTCAGGTCGAGGTGGCCCG | *pilY1-2* deletion |
| *pilY1-2*-BA-FP | ACCCAAGTACCGCCACCTAACCGATGAGGACAAGACAGAA | *pilY1-2* deletion |
| *pilY1-2*-BA-RP | CTGGTAACTGGGAATGGCGAT | *pilY1-2* deletion |
| *pilE*-FA-FP | TCACAAACAGATGAGCGAACA | *pilE* deletion |
| *pilE*-FA-RP | CGTGCCTTCATCCGTTTCCCTCATCGGGGCTGCTCCT | *pilE* deletion |
| *pilE*-Tet^R^-FP | AGGAGCAGCCCCGATGAGATGATTCTCATGTTTGACAGCTT | *pilE* deletion |
| *pilE*-Tet^R^-RP | GTAGAGGGGCTCTTTCGTTTCAGGTCGAGGTGGCCCG | *pilE* deletion |
| *pilE*-BA-FP | ACCCAAGTACCGCCACCTAAAACGAAAGAGCCCCTCTAC | *pilE* deletion |
| *pilE*-BA-RP | GGGCGGGAGGAGAACATTACC | *pilE* deletion |
| *hptC*-FA-FP | CAGCATCAACCAGCAGAGCCG | *hptC* deletion |
| *hptC*-FA-RP | CGTGCCTTCATCCGTTTCCGGGATCCTCAGATCAGCTCGT | *hptC* deletion |
| *hptC*-Tet^R^-FP | ACGAGCTGATCTGAGGATCCCATGATTCTCATGTTTGACAGCTT | *hptC* deletion |
| *hptC*-Tet^R^-RP | TGGACGAAGGCCCGCAACGTCAGGTCGAGGTGGCCCG | *hptC* deletion |
| *hptC*-BA-FP | ACCCAAGTACCGCCACCTAACGTTGCGGGCCTTCGTCCA | *hptC* deletion |
| *hptC*-BA-RP | GCTTTCCGCTGAAGATGGGGC | *hptC* deletion |
| *cupB1*-FA-FP | TGGCTGTTGTAGGAAATGGAC | *cupB1* deletion |
| *cupB1*-FA-RP | CGTGCCTTCATCCGTTTCCCTGATTTCCTTTGGAGTTGTG | *cupB1* deletion |
| *cupB1*-Tet^R^-FP | CACAACTCCAAAGGAAATCAGATGATTCTCATGTTTGACAGCTT | *cupB1* deletion |
| *cupB1*-Tet^R^-RP | CGTCCGTCGACCGCCGCTCAGGTCGAGGTGGCCCG | *cupB1* deletion |
| *cupB1*-BA-FP | ACCCAAGTACCGCCACCTAAGCGGCGGTCGACGGACG | *cupB1* deletion |
| *cupB1*-BA-RP | GGCGTGACACTGAAGGGAACC | *cupB1* deletion |
| *cupC1*-FA-FP | GAGGCGCTATCGAAGCTGATG | *cupC1* deletion |
| *cupC1*-FA-RP | CGTGCCTTCATCCGTTTCCGATTGAGCTTCCTTTTGACAG | *cupC1* deletion |
| *cupC1*-Tet^R^-FP | CTGTCAAAAGGAAGCTCAATCATGATTCTCATGTTTGACAGCTT | *cupC1* deletion |
| *cupC1*-Tet^R^-RP | CCCCGCCCATGTAGCAGAACTCAGGTCGAGGTGGCCCG | *cupC1* deletion |
| *cupC1*-BA-FP | TGCCCCATTATCAGCCTCTAC | *cupC1* deletion |
| *cupC1*-BA-RP | TGCCCCATTATCAGCCTCTAC | *cupC1* deletion |

**Extended data Table 1.** The Strains, plasmids and primers used in this study.

**Part 1.** The different genotype of *P. aeruginosa* strains used in this study, and the competent cells of *E. coli* strains used for gene expression and plasmid amplification. **Part 2.** The list of plasmids constructed in this study, and the description of each vector was indicated in the table. **Part 3.** The list of primers used in this study, and the purpose of each primer was indicated in the table.
