# Supplementary material for: Pseudomonas aeruginosa two-component system LadS/PA0034 regulates macrophage phagocytosis via fimbrial protein cupA1
Source: mBio. 2024 May 21;15(6):e00616-24. doi: 10.1128/mbio.00616-24 (PMC11237798; doi:10.1128/mbio.00616-24)
Supplement: Table S2 — The RNA-seq analysis of pilus genes. [file mbio.00616-24-s0009.docx]

| **Gene_ID** | **count_PA0034.K0** | **count_PAO1** | **fpkm_PA0034.K0** | **fpkm_PAO1** | **log2FC** | **PValue** | **FDR** | **Gene_Name** | **description** |
| --- | --- | --- | --- | --- | --- | --- | --- | --- | --- |
| *PA0992* | 72 | 254.3333 | 35.44333 | 123.37 | -1.8411 | 1.03E-20 | 5.28E-19 | *cupC1* | fimbrial subunit CupC1 |
| *PA0993* | 5.666667 | 22.33333 | 2.24 | 8.716667 | -1.9705 | 2.16E-05 | 0.0003045 | *cupC2* | chaperone CupC2 |
| *PA0994* | 153.3333 | 231.6667 | 12.79 | 19.26333 | -0.5993 | 0.00063544 | 0.0062305 | *cupC3* | usher CupC3 |
| *PA2128* | 96.3333 | 2651.33 | 57.10333 | 1517.17 | -4.7853 | 3.90E-179 | 3.53E-176 | *cupA1* | fimbrial subunit CupA1 |
| *PA2129* | 8.666667 | 474 | 3.206667 | 171.98 | -5.7629 | 4.50E-121 | 2.13E-118 | *cupA2* | chaperone CupA2 |
| *PA2130* | 18.66667 | 602 | 1.493333 | 47.79667 | -5.0028 | 3.39E-117 | 1.23E-114 | *cupA3* | usher CupA3 |
| *PA2131* | 9.666667 | 222.6667 | 1.633333 | 37.43667 | -4.5205 | 7.37E-70 | 1.18E-67 | *cupA4* | fimbrial subunit CupA4 |
| *PA2132* | 6 | 81.66667 | 2.353333 | 31.20667 | -3.7372 | 9.27E-27 | 6.14E-25 | *cupA5* | chaperone CupA5 |
| *PA2133* | 5 | 111.6667 | 1.526667 | 33.69333 | -4.4615 | 2.14E-42 | 2.04E-40 | *PA2133* | hypothetical protein |
| *PA4081* | 27 | 68 | 5.616667 | 13.93 | -1.3284 | 5.16E-07 | 9.64E-06 | *cupB6* | fimbrial subunit CupB6 |
| *PA4082* | 107.3333 | 329 | 7.25 | 21.99333 | -1.6108 | 1.99E-19 | 9.49E-18 | *cupB5* | adhesive protein CupB5 |
| *PA4083* | 4 | 7.333333 | 1.496667 | 2.66 | -0.8608 | 0.17294476 | 0.4197658 | *cupB4* | chaperone CupB4 |
| *PA4084* | 21.33333 | 70.33333 | 1.763333 | 5.886667 | -1.7335 | 3.48E-10 | 9.26E-09 | *cupB3* | usher CupB3 |
| *PA4085* | 4.333333 | 48.66667 | 1.613333 | 17.83333 | -3.4736 | 6.82E-17 | 2.81E-15 | *cupB2* | chaperone CupB2 |
| *PA4086* | 29.33333 | 253 | 16.51333 | 137.8667 | -3.1119 | 2.92E-46 | 3.11E-44 | *cupB1* | fimbrial subunit CupB1 |
| *PA4550* | 174 | 520 | 119.5533 | 344.54 | -1.5844 | 1.90E-22 | 1.08E-20 | *fimU* | type 4 fimbrial biogenesis protein FimU |
| *PA4551* | 143.333 | 413 | 83.41 | 233.0733 | -1.5351 | 5.85E-22 | 3.24E-20 | *pilV* | type 4 fimbrial biogenesis protein PilV |
| *PA4552* | 201.667 | 558.667 | 64.69333 | 176.9333 | -1.4826 | 8.12E-21 | 4.20E-19 | *pilW* | type 4 fimbrial biogenesis protein PilW |
| *PA4553* | 114.333 | 347.333 | 60.98667 | 179.2867 | -1.6052 | 3.99E-20 | 1.97E-18 | *pilX* | type 4 fimbrial biogenesis protein PilX |
| *PA4554* | 2463.33 | 5204.67 | 144.5167 | 307.4833 | -1.0982 | 2.17E-17 | 9.21E-16 | *pilY1* | type 4 fimbrial biogenesis protein PilY1 |
| *PA4555* | 111.667 | 269 | 172.0467 | 383.4933 | -1.2665 | 8.12E-11 | 2.27E-09 | *pilY2* | type 4 fimbrial biogenesis protein PilY2 |
| *PA4556* | 288.667 | 653.333 | 278.26 | 597.9967 | -1.1823 | 1.26E-13 | 4.19E-12 | *pilE* | type 4 fimbrial biogenesis protein PilE |

Extended data Table 2

**Extended data Table 2.** The reduced expression of pilus genes in △*PA0034* compared to PAO1 strains, data from RNA-seq analysis.
